# Domain function and predicted structure of three heterodimeric endonuclease subunits of RNA editing catalytic complexes in *Trypanosoma brucei*

**DOI:** 10.1093/nar/gkac753

**Published:** 2022-09-12

**Authors:** Jason Carnes, Suzanne M McDermott, Isaac Lewis, Maxwell Tracy, Kenneth Stuart

**Affiliations:** Seattle Children's Research Institute, Seattle, WA, USA; Seattle Children's Research Institute, Seattle, WA, USA; Department of Pediatrics, University of Washington School of Medicine, Seattle, WA, USA; Seattle Children's Research Institute, Seattle, WA, USA; Seattle Children's Research Institute, Seattle, WA, USA; Seattle Children's Research Institute, Seattle, WA, USA; Department of Pediatrics, University of Washington School of Medicine, Seattle, WA, USA

## Abstract

Each of the three similar RNA Editing Catalytic Complexes (RECCs) that perform gRNA-directed uridine insertion and deletion during *Trypanosoma brucei* mitochondrial (mt) mRNA editing has a distinct endonuclease activity that requires two related RNase III proteins, with only one competent for catalysis. We identified multiple loss-of-function mutations in the RNase III and other motifs of the non-catalytic KREPB6, KREPB7, and KREPB8 components by random mutagenesis and screening. These mutations had various effects on growth, editing, and both the abundances and RECC associations of these RNase III protein pairs in bloodstream form (BF) and procyclic form (PF) cells. Protein structure modelling predicted that the Zinc Finger (ZnF) of each paired RNase III protein contacts RNA positioned at the heterodimeric active site which is flanked by helices of a novel RNase III-Associated Motif (RAM). The results indicate that the protein domains of the non-catalytic subunits function together in RECC integrity, substrate binding, and editing site recognition during the multistep RNA editing process. Additionally, several mutants display distinct functional consequences in different life cycle stages. These results highlight the complementary roles of protein pairs and three RECCs within the complicated *T. brucei* mRNA editing machinery that matures mt mRNAs differentially between developmental stages.

## INTRODUCTION

Mitochondrial (mt) gene expression in *Trypanosoma brucei* entails extensive RNA editing, the guide RNA (gRNA) directed insertion and deletion of uridines (Us) that converts mt transcripts into translatable mRNAs for respiratory chain components (1–4). The 12 mitochondrial transcripts that undergo RNA editing have more than a thousand distinct editing sites (ESs) that are specified by gRNAs (5,6). In addition, some mRNAs are differentially edited between life cycle stages, which coincides with different compositions and functions of energy generation systems throughout the parasite life cycle. Procyclic form (PF) parasites utilize oxidative phosphorylation for ATP generation within the insect vector and rely on editing of mRNAs that encode multiple proteins of the respiratory chain, e.g. cytochromes (7–11). In contrast, bloodstream form (BF) parasites within the mammalian host lack cytochrome activity, preferentially edit the 3′ portion of the ND7 component of respiratory complex I, employ glycolysis for ATP production, and use ATP synthase to hydrolyse ATP and thereby maintain essential mitochondrial membrane potential (12,13). The mechanisms responsible for the developmental changes in RNA editing have long been elusive, but recent insights into life cycle-dependent functionality of RNA Editing Catalytic Complexes (RECCs) are advancing understanding of this phenomenon (14–17).

RECCs, previously called ∼20S editosomes, are multiprotein complexes that catalyse coordinated endonucleolytic cleavage, U insertion or deletion, and ligation at ESs of mitochondrial mRNAs which are duplexed with cognate gRNAs (4) (see Supplementary Table S1 and (4) for full terminology and abbreviations of RECC proteins). Three functionally distinct RECCs contain mutually exclusive pairs of endonucleases and partner proteins: KREN3/KREPB6 (N3/B6), KREN2/KREPB7 (N2/B7), and KREN1/KREPB8 (N1/B8). N1/B8 RECCs also uniquely contain the U specific KREX1 (X1) exonuclease. In addition, all three RECCs have a common set of 12 proteins including those with U addition, U removal, and RNA ligation capabilities and the others that have no known or apparent catalytic capability (18–22). N1/B8/X1 RECCs perform deletion editing, while N2/B7 and N3/B6 RECCs perform insertion editing with distinct preferences for different ESs (19,21,23,24). Importantly, nearly half of RECC proteins, including the endonucleases and their partners, are paralogs that contain ZnF and RNase III motifs. The N1, N2 and N3 endonucleases contain catalytically functional RNase III motifs but their respective B8, B7 and B6 partners have RNase III motifs that lack key catalytic residues. While RNase III dimers in many other endonucleases cleave both RNA strands, the catalytic and non-catalytic RNase III dimer arrangement in RECCs may limit cleavage to the mRNA of the mRNA/gRNA heteroduplex (25–28). Each RECC has a single copy of either N1, N2, or N3 (20) in close proximity to their respective non-catalytic B8, B7 or B6 partner, as shown by cross-linking mass spectrometry (22). Furthermore, the total abundances of the endonucleases are dramatically reduced upon loss or RNase III motif mutation of their partner protein (15). This evidence indicates that N1, N2 and N3 form heterodimers with their non-catalytic RECC partners resulting in an active catalytic site analogous to other RNase III endonucleases all of which have dimeric RNase III domains (28). These pairings of RECC RNase III proteins may therefore also enable non-catalytic pseudoenzyme regulation of the catalytic component (15,29–34). Because cleavage is the first step in the coordinated catalytic cycle at each ES, endonuclease recognition could play an important role in regulating RNA editing, including differentially between life cycle stages. Although RECC protein composition appears to be essentially the same in BF and PF cells (20), random and site-specific mutagenesis studies of B5 and A3 identified single amino acid changes with different consequences in BF versus PF cells. These life cycle-dependent differences suggest that RECCs function differentially between life cycle stages despite the lack of apparent differences in their protein composition (14–17).

We report here the analysis of BF and PF *T. brucei* that have loss of function amino acid (aa) substitutions in B6, B7 or B8 which were identified in conditional null (CN) BF cell lines that exclusively express randomly mutagenized genes. Mutations that mapped to RNase III, ZnF, novel RNase III associated motifs (RAM) or elsewhere in the proteins had various consequences to the cellular abundances and associations with RECCs of these proteins and their cognate endonucleases, and to RNA editing, including differential effects between parasite life cycle stages. Molecular modelling predicts that B6/N3, B7/N2 and B8/N1 are heterodimers in which the RNase III domains are paired, with each pair having a single catalytic site. These paired RNase III domains are flanked by RAM α-helices (one from each protein), and the ZnFs of each protein interact with RNA positioned across the active site. This arrangement provides for cleavage of one strand of the mRNA/gRNA heteroduplex, i.e. the mRNA, and its positioning for subsequent catalytic steps. The functional consequences of the mutations are consistent with these structures and indicate roles of the protein domains in the multiple events that must be performed by RECCs during editing, including steps that differ between BF and PF life cycle stages.

## MATERIALS AND METHODS

### Growth of cells in vitro

BF cells were grown in HMI-9 (35) with 10% FBS at 37°C, 5% CO_2_. PF cells were grown in SDM-79 (36) with 10% FBS at 27°C, both supplemented with 0.5 μg/ml tet where appropriate, unless otherwise stated. For growth curve analysis, cell density was measured using a Coulter Counter. BF cells were reseeded at 1 to 2 × 10^5^ cells/ml in 10 ml every day, whilst PF cells were reseeded at 2 × 10^6^ cells/ml in 10 ml every two days. To generate cell growth heat maps, cumulative growth numbers for –tet and + tet cultures were calculated for each time point. Next, log_2_ of the ratio of –tet to +tet was calculated, and this number was converted to blue to orange scale using conditional formatting in Microsoft Excel.

### Error-prone PCR to generate mutant libraries for transfection

Error-prone PCR mediated mutagenesis of B6, B7 and B8 was performed using the approach previously described for B5 (16). Briefly, wild-type (WT) open reading frames (ORFs) for B6 and B7 lacking stop codons, or B8 containing a stop codon were flanked by *att*B1 (5′) and *att*B2 (3′) sites in pHD1344tub(PAC)-B6-Cterm3V5, pHD1344tub(PAC)-B7-Cterm3V5 or pHD1344tub(PAC)-Nterm3V5-B8, respectively. 100 ng of each ORF was mutagenized by 20 or 30 cycles of PCR using GeneMorph II Random Mutagenesis kit (Agilent Technologies) according to the manufacturer's protocol with the following primers: attB1 5′-ACAAGTTTGTACAAAAAAGCAG-3′, attB2 5′-ACCACTTTGTACAAGAAAGCT-3′. Mutagenized ORFs were gel-purified and transferred into pDONR-Express using BP Clonase II (Life Technologies) followed by transformation into TOP10 Electrocompetent cells (Life Technologies) as previously described (16). To obtain full-length B6, B7, or B8 alleles, an aliquant of the transformation was serially diluted and plated to titer the number of Kan^+^ colonies, while the remainder of the transformation was stored as a glycerol stock. The optimal kanamycin concentration for selection of full-length B6, B7 and B8 in the pDONR-Express system was determined to be 10, 10 and 15 μg/ml respectively. After determining titers, glycerol stocks were thawed on ice and plated out to a density of ∼1000 colonies/plate to produce an overall number of ∼40 000 Kan^+^ colonies. After 36 h incubation at 30°C, all colonies were harvested, and plasmid DNA was isolated using the QIAfilter midiprep kit (Qiagen). These plasmid libraries of full-length mutant B6, B7, or B8 alleles are termed pENTR-Express allele libraries. 250 ng of each pENTR-Express mutant allele library was combined with 500 ng of the destination vector pHD1344tub(PAC)GW-Cterm3V5 for B6 and B7 (14), 2 μl of LR Clonase II enzyme mixture (Life Technologies), and TE to 10 μl were incubated at room temperature (25°C) for 20 h. For B8, the destination vector was pHD1344tub(PAC)GW-Nterm3V5 (37). Reactions were stopped by adding Proteinase K (1 μl/10 μl of reaction volume) and incubating at 37°C for 10 min. These LR reactions generate expression vectors pHD1344tub(PAC)-mutB6-Cterm3V5, pHD1344tub(PAC)-mutB7-Cterm3V5 and pHD1344tub(PAC)-Nterm3V5-mutB8, which contain the puromycin resistance (*PAC*) selectable marker and constitutively expression 3×V5 tagged mutant library alleles from the *β-tubulin* locus. LR reactions were transformed into TOP10 Electrocompetent cells (Life Technologies), serially diluted and titered as described above. After determining titers, glycerol stocks were thawed on ice and plated to a density of 1000 colonies/plate on LB/Amp (100 μg/ml) plates to produce ∼40 000 Amp^+^ colonies. After 36 h incubation at 30°C, all colonies were harvested, and plasmid DNA was isolated using the QIAfilter midiprep kit (Qiagen). These expression plasmid libraries of mutant B6, B7 or B8 alleles were subsequently used to transfect corresponding BF Conditional Null (CN) cell lines as described below.

### Transfection and complementation screening of mutant allele libraries

Transfections of mutant allele libraries and complementation screening of B6, B7 and B8 were performed using the approach previously described for B5 (16). Throughout transfection and screening, unless otherwise stated, all cell lines were grown in 5 ng/ml tet, which is the minimum concentration of tet required for cell growth at wild-type level. For each screen, five replicate transfections were performed, each using 10 μg of *Not*I-linearized expression plasmid library for B6, B7, or B8 alleles (described above) into 3 × 10^7^ BF cells in a volume of 100 μl using the Amaxa nucleofector (Lonza) (38). The appropriate libraries were transfected into BF B6 CN, B7 CN, or B8 CN cell lines (15). Following transfection, cells were diluted into 300 mL HMI-9 medium containing 2.5 μg/ml G418, 5 μg/ml hygromycin, 2.5 μg/ml phleomycin, and 5 ng/ml tet, and 1 ml plated per well in 24-well plates. Cells were allowed to recover for 24 h before selection in 0.1 μg/ml puromycin. After five days of selection, a total of 833 B6, 835 B7 and 933 B8 puromycin-resistant cell lines were isolated and arrayed in 96-well plates containing 200 μl HMI-9 medium containing 2.5 μg/ml G418, 5 μg/ml hygromycin, 2.5 μg/ml phleomycin, 0.1 μg/ml puromycin and 5 ng/ml tet and grown to a density of approximately 2 × 10^5^ cells/ml. Transfection efficiencies were 5.6 × 10^–5^ for B6, 5.1 × 10^–5^ for B7, and 6.2 × 10^–5^ for B8. Replica plates made by transferring 2 μl cells into 200 μl HMI-9 medium that either contained 5 ng/ml tet or completely lacked tet were then used to assess cell growth. Dilution into medium lacking tet reduces tet concentration below the minimum required for cell growth in parental BF CN cells. Cells with or without tet were grown for three days, then 2 μl cells re-plated into 200 μl HMI-to maintain logarithmic growth and grown for an additional three days. Cell viability was then assessed by incubating with 20 μl alamarBlue (Life Technologies) for 4 h, which results in a colorimetric change (dark blue changes to bright pink in the presence of proliferating cells) and a fluorescent signal in response to metabolic activity (39). Fluorescence was measured using a SpectraMax M2 microplate reader (Molecular Devices) with excitation at 544 nm and emission at 590 nm. Cells that survive in the presence of tet but have a growth defect in the absence of tet contain a mutant allele that could not complement the loss of tet-regulated WT expression in the CN background. Moderate or severe growth defects in the absence of tet were identified in 215 B6, 86 B7 and 275 B8 cell lines, respectively.

### Identification of library alleles by PCR amplification and sequencing

DNA extraction, PCR amplification, purification, and sequencing were carried out in 96 well format using the approach previously described for B5 (16). Primer sequences are listed in Supplementary Table S2. Briefly, ∼2 × 10^4^ cells in 10 μl were lysed in 20 μl Lysis Solution for Blood (Sigma-Aldrich) at 75°C for 5 min, 180 μl Neutralization Solution for Blood (Sigma-Aldrich) added, and crude DNA samples stored at 4°C. Library alleles in the *β-tubulin* locus were PCR amplified with Phusion high-fidelity polymerase (NEB) in two steps; first using 2.5 μl of each crude DNA sample with forward primer (5355) that anneals 5′ of the plasmid integration site in the genomic *β-tubulin* locus, and reverse primer (6931) in the coding sequence for the C-terminal V5-tag for B6 and B7. Next, B6 and B7 sequences were PCR amplified using nested primers 9571 and 10622 to obtain PCR products for sequencing as previously described (16). B6 and B7 PCR products were sequenced using primers 11316 and 11317 to obtain the entire coding sequence. To PCR amplify B8, which has an N-terminal V5-tag, primers 5355 and 10150 were used for first step PCR amplification, and then nested primers 9571 and 5356 were used to obtain PCR products for sequencing. B8 PCR products were sequenced using the same primers (9571 and 5356).

### Generation of exclusive expression cell lines by site-directed mutagenesis

For BF transfections, WT expression plasmids pHD1344tub(PAC)-B6-Cterm3V5, pHD1344tub(PAC)-B7-Cterm3V5 or pHD1344tub(PAC)-Nterm3V5-B8 (15) were used as a template for site-directed mutagenesis (QuikChange II kit; Agilent) using forward and reverse primers listed in Supplementary Table S2. For PF transfections, the PAC cassette was replaced with NAT selectable marker. *Not*I-linearized plasmids were transfected into the corresponding BF or PF B6, B7 or B8 CN cell line that contains cognate endonuclease that is HA-tagged at its endogenous locus as previously described (15) in the presence of 0.5 μg/ml tet (38). BF cell lines resistant to 0.1 μg/ml puromycin and PF cells resistant to 100 μg/ml nourseothricin were selected, and constitutive expression of B6-3xV5, B7-3xV5 or 3xV5-B8 was confirmed by Western blot.

### RT-PCR analysis of RNA editing profiles

Total RNA was harvested using TRIzol and treated with TURBO DNase (Life Technologies) according to manufacturer's instructions from cells grown either 2 days (BF) or 4 days (PF) after tet withdrawal to avoid secondary effects from growth defects. RNA integrity was confirmed by RNA nanochip on a BioAnalyzer (Agilent Technologies). cDNAs were made by target-specific priming using oligos that anneal to never-edited 3′ regions of A6, MURF2, RPS12, ND7-5′, ND8, CYb and never-edited ND4 control (Supplementary Table S2). For RT-PCR reactions, 35 cycles of amplification were performed for all targets except CYb, which used 25 cycles of amplification, and products were resolved on 3% agarose TBE gels, stained with ethidium bromide, and visualized using AlphaImager EP (AlphaInnotech). Cycling conditions were tested to ensure that 35 cycles of amplification did not introduce artifacts (Supplementary Figure S1).

### BN-PAGE and Western analysis

For both BF and PF experiments, cell lysates for BN-PAGE were created by harvesting 1 × 10^8^ cells, resuspending in 225 μl IPP150 lysis buffer (10 mM Tris–HCl pH 8.0, 150 mM NaCl, 0.1% Nonidet P-40) plus cOmplete protease inhibitors (Roche, as directed by manufacturer), then adding 25 μl 10% Triton X-100. Cells were harvested at either 2 days (BF) or 4 days (PF) after tet withdrawal to avoid secondary effects from growth defects. Cells were lysed by rotating at 4°C for 20 minutes and clarified by centrifugation at 12 000 × g for 10 minutes at 4°C. 37.5 μl of cleared lysate was added to 15 μl sample load buffer [a combination of 12.5 μl of 4× NativePAGE Sample Buffer and 2.5 μl NativePAGE 5% G-250 sample additive (Thermo Fisher Life Tech)]. Of this, 10.5 μl was resolved on NativePAGE Bis-Tris 3–12% gels (Thermo Fisher Life Tech). NativeMark unstained protein standard (Thermo Fisher Life Tech) was used as a size reference. Proteins were transferred onto Immobilon-P (Millipore) PVDF membrane and fixed by incubation in 8% acetic acid for 15 min at room temperature. Blots were probed either with V5 epitope tag monoclonal antibody (1:5,000 dilution; ThermoFisher Scientific), HA epitope tag monoclonal antibody (2–2.1.14) (1:2000 dilution; ThermoFisher Scientific), or 1:12.5 dilution of anti-KREPA2 monoclonal antibody (40) (Supplementary Table S3). Blots were developed with an enhanced chemiluminescence kit (Thermo Scientific) per the manufacturer's instructions and imaged using the FluorChem E system (ProteinSimple) or x-ray film (McKesson).

### Multiple sequence alignments and sequence identity heat maps

Multiple sequence alignments were performed with Geneious software (version 2019.1.1) using MUSCLE (version 3.8.425) algorithm with default parameters. Amino acid sequences for N1, N2, N3, B4, B5, B6, B7 and B8 were obtained from TriTrypDB (41) (Supplementary Table S4). Sequences were taken from a wide phylogenetic distribution of species to perform alignments of RECC RNase III proteins. To create percent identity heat maps, the percent identity at a position in the alignment was mapped to corresponding amino acid in the *T. brucei* sequence and this number was converted to yellow to magenta scale using conditional formatting in Microsoft Excel.

### Structural predictions and protein structure modelling

Predicted structures for the *T. cruzi* orthologs of B6 (Q4CPS4), B7 (Q4DHG3), B8 (Q4CX36), N1 (Q4DA91), N2 (Q4E082) and N3 (Q4E095) were obtained from the Alphafold Protein Structure Database (https://alphafold.ebi.ac.uk/) (42). Unstructured and disordered regions of very low (AlphaFold pLDDT score < 50) and low (AlphaFold pLDDT score < 70) confidence at the N- and C-termini of each protein were removed for clarity. Predicted structures were modeled onto *Saccharomyces cerevisiae* Rnt1p and *Aquifex aeolicus* RNase III crystal structures (5T16 and 2NUF respectively) using the Matchmaker function in Chimera (43) and ChimeraX (44). Crosslink visualization and distance measurements on structures were also performed using Chimera.

## RESULTS

### Random mutagenesis libraries and complementation screening

**Figure 1.**
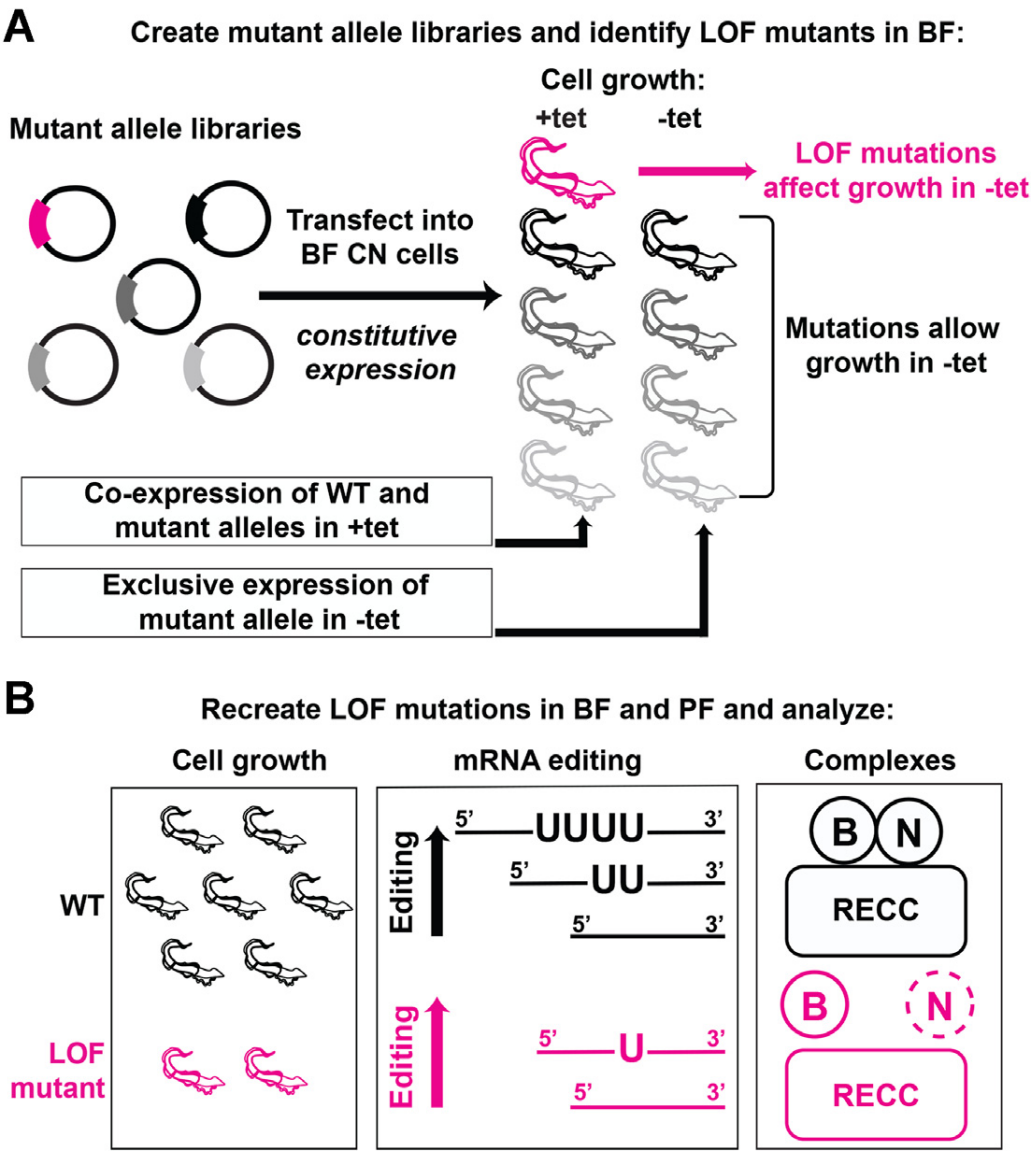
Schematic of random mutagenesis and complementation screen. (**A**) B6, B7 and B8 mutant allele libraries were generated by error-prone PCR and transfected into bloodstream form (BF) conditional null (CN) cell lines for constitutive expression. CN cells require tetracycline (tet) for expression of a regulatable WT allele. Removal of tet results in exclusive expression of mutant alleles enabling identification of loss of function (LOF) mutations as cell growth defects. (**B**) Alleles containing LOF mutations identified in (A) were recreated by site-directed mutagenesis and transfected into BF and procyclic form (PF) CN cell lines for further analyses of effects on growth, editing, and RECCs in BF versus PF.

**Figure 2.**
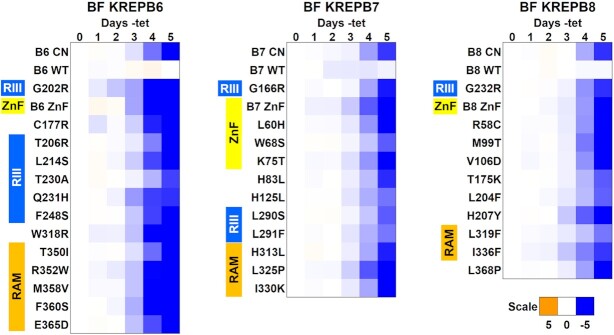
Effect on growth of exclusive expression of alleles with a mutated single amino acid. The log2 ratio of the effect on cumulative growth in –tet versus +tet is indicated by the scale showing reduction in blue, increase in orange, and no effect on growth in white. Growth of parental CN cells are at the top and above that of cells exclusively expressing wild-type (WT) or mutant alleles as indicated, including B6 (G202R), B7 (G166R) and B8 (G232R) which disrupt function (15) as controls. Data are representative of a minimum of three experiments.

We generated libraries of ∼40 000 plasmids containing mutated full length B6, B7 and B8 alleles by error-prone PCR. Sequencing tens of random clones showed that the allele libraries contained a range of mutations, and that each library had at least 10 000 alleles with single aa changes (Supplementary Figure S2 and Supplementary Table S5). The allele libraries were transferred into our *T. brucei* destination vectors pHD1344tub(PAC)GW-Cterm3V5 for B6 and B7, or pHD1344tub(PAC)GW-Nterm3V5 for B8, and transfected into their respective CN *T. brucei* cell lines. For each library we isolated close to 1,000 cell lines, of which 11% (B7), 26% (B6) and 29% (B8) displayed growth defects upon exclusive expression of their mutant alleles (Figure 1 and Supplementary Table S6). We sequenced these non-complementing mutant alleles, focusing specifically on the identification of those containing single aa substitutions to simplify deconvolution of mutant phenotypes (Figure 1 and Supplementary Table S7). We confirmed that the single aa changes were responsible for the growth defects observed in our screens by recreating site-directed V5-tagged mutant constructs and transfecting them into the corresponding BF CN cell lines that also contain endogenously HA-tagged cognate endonucleases (15). Because the ZnF is essential in the related protein B4, we also generated B6–B8 cell lines in which conserved ZnF cysteines were mutated to alanines. Exclusive expression of all these mutant alleles resulted in growth phenotypes due to the single aa or ZnF changes (Figure 2). Growth of the parental CN cell lines, cell lines expressing V5-tagged WT alleles, and cells expressing V5-tagged glycine to arginine (G→R) RNase III mutant alleles that had been previously characterized were also included for comparison (15). Thus, we identified and validated several single aa or ZnF mutations in B6, B7 or B8 that impacted function.

**Figure 3.**
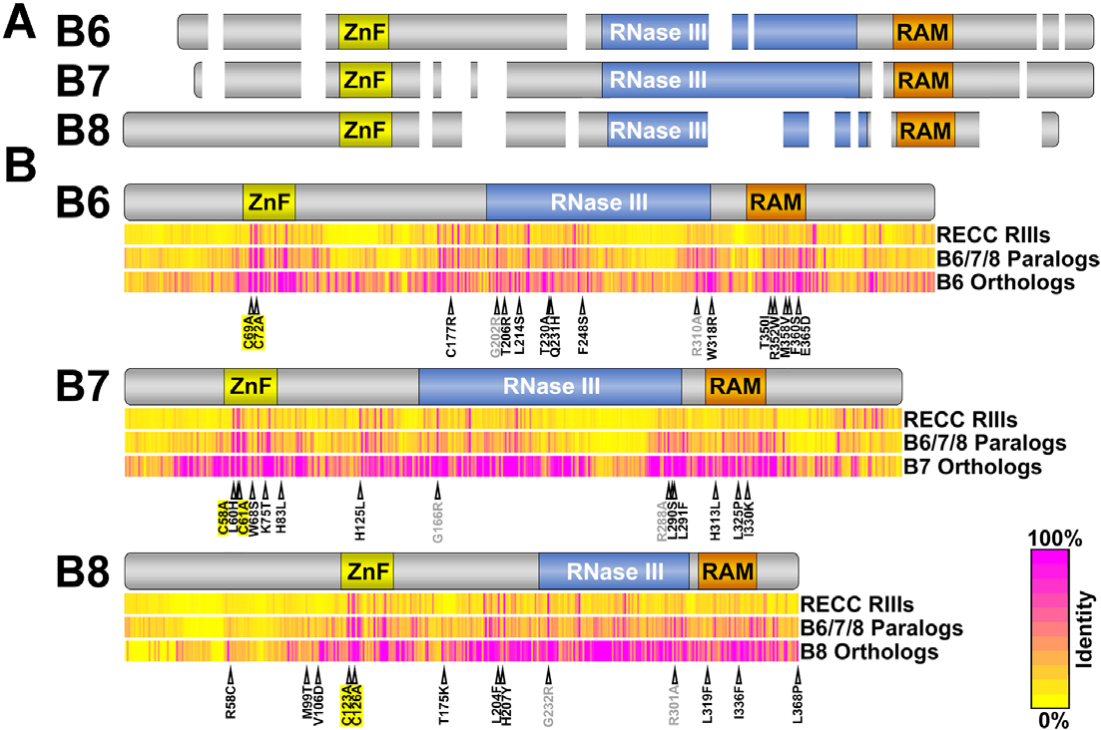
Alignments of B6, B7 and B8 motifs, amino acid sequences and positions of loss-of-function mutations. (**A**) Schematic alignment of *T. brucei* B6, B7 and B8 amino acid sequences showing gaps larger than two residues with the zinc-finger (ZnF), RNase III and RNase III-Associated Motif (RAM) regions indicated. The RNase III motif boundaries are from (22) and those for ZnF and RAM are established herein. (**B**) Schematic of B6, B7 and B8 with regions of conservation indicated according to the heat map scale of % identity. The three multispecies alignments show: (top) all RECC RNase III endonuclease paralogs and orthologs of N1, N2, N3, B4, B5, B6, B7 and B8; (middle) the B6, B7 and B8 paralogs and orthologs; (bottom) the orthologs of B6, B7 or B8. The positions of loss of function single amino acid mutations identified here are indicated in black, published mutations are in gray (15), and double ZnF mutations are highlighted in yellow.

We localized these mutations in B6-B8 relative to annotated motifs and regions of aa sequence conservation (Figure 3 and Supplementary Tables S8-S10). Alignment of *T. brucei* B6-B8 illustrates greater sequence similarity between B6 and B7 versus B8 and shows that B8 contains N-terminal sequences that are not in B6 or B7 and in which we identified mutations that disrupt B8 function (M99T and V106D) (Figure 3A). Because N1 - N3 and B4 - B8 are paralogs that contain both ZnF and RNase III motifs (Supplementary Tables S1 and S4) we also identified regions of sequence conservation among phylogenetically diverse kinetoplastids that are 1) within all B6, B7 or B8 orthologs, 2) among all kinetoplastid B6-B8 paralog sequences, or 3) among all N1–N3 and B4–B8 sequences (Figure 3B). The comparisons within and between B6-B8 orthologs and their paralogs show conservation in the previously annotated ZnF and RNase III motifs (Figure 3B). They also reveal a previously unrecognized region of high sequence conservation C-terminal to the RNase III motif in B6-B8, which we call the RNase III-Associated Motif (RAM). Many of our validated single aa substitutions are within the previously annotated RNase III and ZnF motifs and the newly identified RAM. The substitutions were generally non-conservative with different properties from the original aa (Supplementary Table S7). The B6 (T350I, R352W, M358V, F360S and E365D), B7 (H313L, L325P, and I330K), and B8 (L319F and I336F) substitutions in the RAM were clustered in a region that aligns with a region in N1–N3 and B4–B5 paralogs previously identified as a PUF motif (14,15,22,45) (Supplementary Figure S3).

### Life cycle-dependent differences in B6, B7 and B8 function

**Figure 4.**
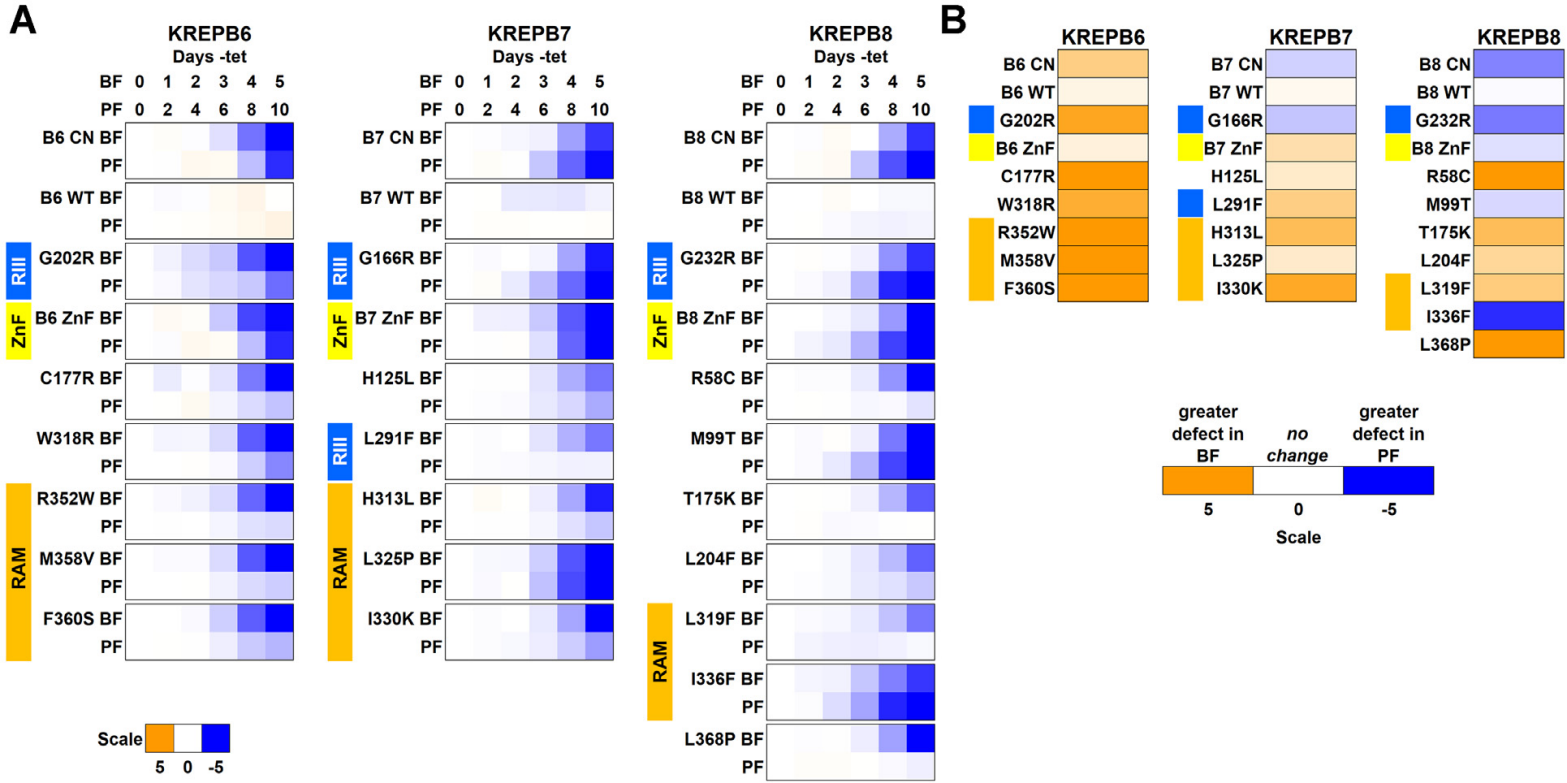
Comparison of BF and PF growth upon exclusive expression of mutated alleles. (**A**) Reduction (blue) or increase (orange) in growth upon knockdown, or exclusive expression of WT or mutated alleles. The time scales are adjusted account for the approximate 2× growth rate of BF versus PF. Color scale as in Figure 2. Data are representative of a minimum of three experiments. (**B**) Relative effect on growth of PF versus BF. Heat map of the log_2_ ratio of cumulative growth in –tet versus +tet for PF on day 10 and BF on day 5.

Having identified mutations that disrupted function of B6, B7 and B8 in BF, we examined the effects of these mutations in PF parasites with a focus on mutations in the conserved ZnF, RNase III motif, and RAM regions, and the R58C, M99T and L368P mutations that were uniquely found in the B8 screen. The selected mutations were introduced into the corresponding PF CN cell lines that contain HA-tagged cognate N1, N2 or N3 endonucleases in the endogenous loci. Cell growth was determined upon exclusive expression of the mutant B6, B7, or B8 alleles relative to the parental CN cell lines and cell lines expressing V5-tagged WT or G→R RNase III mutant alleles (15), and compared to growth of analogous BF lines (Figure 4). Growth was normal in both life cycle stages upon exclusive expression of WT B6, B7 or B8 allele, but loss of regulated WT gene expression in parental CN and exclusive expression of G→R RNase III mutant cells strongly reduced growth in PF as expected (15). Our selected mutations resulted in a range of growth effects in PF versus BF. B6 ZnF, and B7 ZnF, H125L and L325P had similar effects on growth in PF and BF, and B7 G166R, and B8 G232R, ZnF, M99T, and I336F mutations reduced growth in PF even more than observed in BF. B7 H313L and I330K, B8 L204F, and all B6 mutations except ZnF reduced growth in PF to a lesser extent than in BF. In contrast, exclusive expression of B7 L291F, B8 R58C, T175K, L319F and L368P had small or no effects on PF growth, but strong effects on BF growth.

These results highlight differences within B6-B8, where mutations in the same protein motif or region have differential effects on BF and PF growth. For example, the R58C and M99T mutations N-terminal to the ZnF motif in B8 had opposite effects, with the former having almost no effect on PF growth and the latter having a greater effect in PF than BF. They also reveal differences between B6, B7 and B8, where homologous mutations have distinct phenotypes in BF and PF. For example, the B6 R352W, M358V, F360S, and B7 H313L and I330K RAM motif mutations affected growth less in PF than in BF, whereas the B8 L319F RAM mutation did not noticeably affect PF growth and the B7 L325P and the B8 I336F RAM mutations affected PF growth similar to or more than in BFs. Together, these data illustrate the complexity of the functional roles that these proteins and their motifs play within RNA editing, and underscore functional differences between PF and BF RECCs.

### Consequences of B6, B7 and B8 mutations to RNA editing

**Figure 5.**
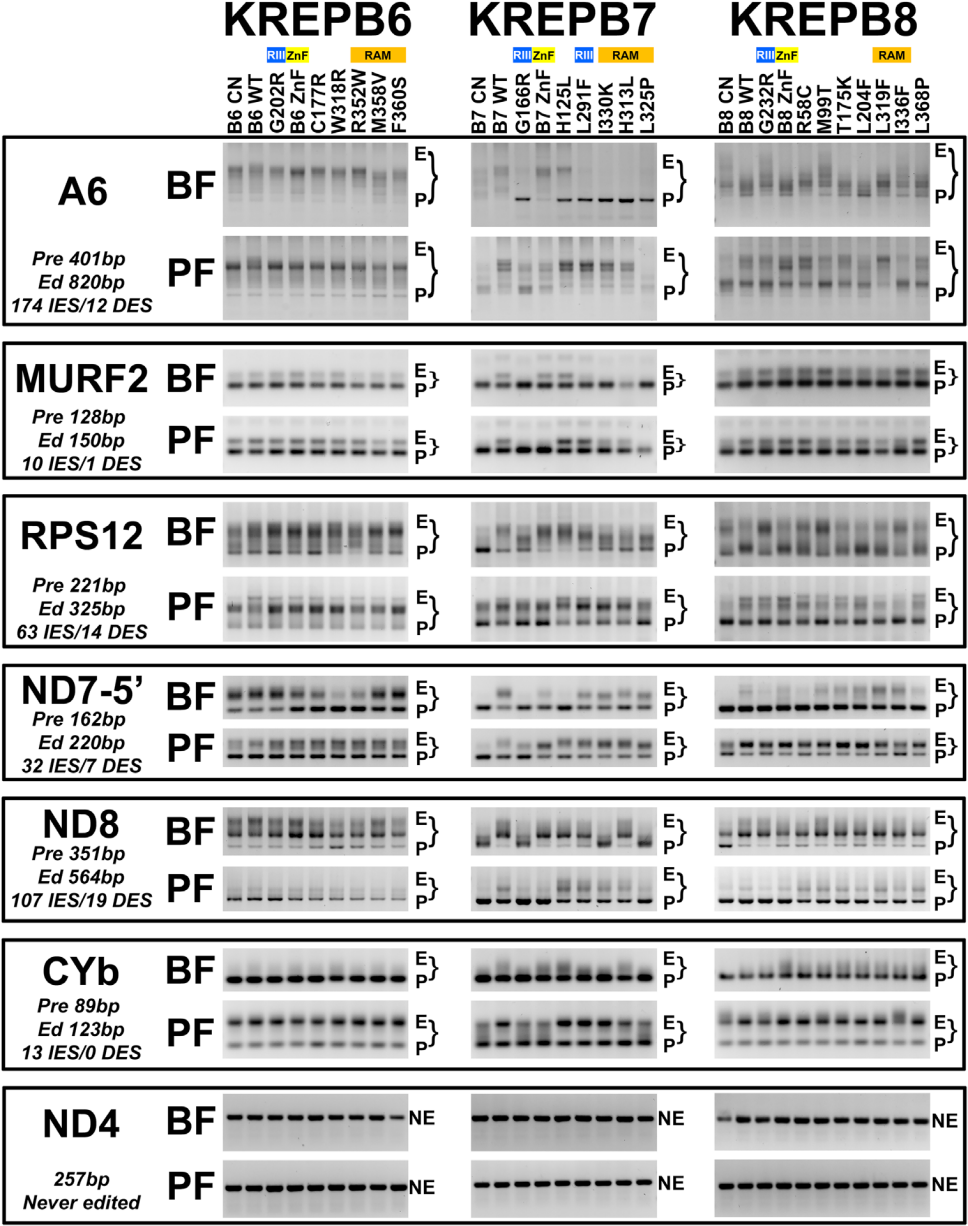
Consequences to mRNA editing upon exclusive expression of mutant alleles in BF or PF as shown by RT-PCR. Brackets indicate gel regions that span the sizes from pre-edited (P) to fully edited (E) RT-PCR products from A6, MURF2, RPS12, ND7-5′, ND8 or CYb mRNAs. Never-edited (NE) ND4 mRNA is included as a mitochondrial mRNA control.

To assess how the various mutations to B6, B7, and B8 affected RNA editing, we examined the profiles of pre-, partially-, and fully- edited mRNAs for A6, MURF2, RPS12, ND7-5′, ND8 and CYb by RT-PCR in both BF and PF CN cell lines that were exclusively expressing mutant alleles (Figure 5). Editing of CYb and ND8 are strongly upregulated in PF and BF respectively, while other transcripts are broadly edited in both life cycle stages (46). In these assays, primers anneal to 5′ and 3′ regions of mRNAs and thereby generate RT-PCR products from all pre-edited, partially edited and fully edited mRNAs. As editing progresses, the sizes of the RT-PCR products become progressively larger because the great majority of ESs are insertion sites, and only a small fraction of the mRNA population is fully edited for some transcripts. The never edited ND4 mRNA is included as a control for RT-PCR of mt mRNAs. While the effects on editing are likely specific to the functions of each B6, B7, and B8 containing RECC, the observed editing includes that done by the other RECCs that are retained and whose expression may increase as is seen upon knockdown of B6, B7 or B8 in CN cells (15,20,23). Furthermore B6/N3 and B7/N2 RECCs have distinct preferences at insertion ESs (IESs), while B8/N1 RECC edits deletion ESs (DESs) (15,21). Thus, the observed RT-PCR editing profiles are the combined result of editing by all three RECCs, with the largest defects resulting in cases where the mutant protein is uniquely required for editing to progress.

ES preferences were especially evident in the differential effects of the various mutations in B6, B7, and B8 on editing of A6 mRNA (174 IESs, 12 DESs), but there were also noticeable effects on editing of the MURF2 (10 IESs, 1 DES), RPS12 (63 IES, 14 DES), ND7-5′ (32 IES, 7 DES), ND8 (107 IES, 19 DES) and CYb (13 IES, 0 DES) mRNAs (Figure 5). Mutations in B7, and to a lesser extent B8, generally resulted in the loss of a wide range of partially- and fully-edited products compared to WT and a relative increase in products with the sizes of pre-edited mRNAs. In contrast, mutations in B6 resulted primarily in loss of only the largest edited products compared to WT, but not of partially edited species. These results are consistent with those previously observed upon knockdown or knockout of B6-B8 and N1-N3 (15,19,23,24). The greater effects on editing in B7 mutants, especially in BFs, reflect loss of editing at many IESs predominantly recognized by B7/N2 and not B6/N3 RECCs. Conversely, the lesser effects of B6 mutants reflect the relatively fewer sites in A6, RPS12, MURF2, ND7-5′, ND8, and CYb, where B6/N3 RECC activity is uniquely required. Similarly, the small consequence of B8 mutations to MURF2 and CYb RT-PCR profiles in BF and PF is because MURF2 has a single DES (−4 Us) and CYb has no DESs. Although CYb does not contain DESs, deletion editing activity appears to be required to produce a normal CYb editing RT-PCR profile. This is based on our observations of CYb sequences that are larger than the expected fully-edited product in multiple PF B8 mutants, most notably I336F. The amount and diversity of A6, RPS12, ND7-5′ and ND8 products across B8 mutants is also consistent with the loss of deletion editing activities, i.e. editing is performed by a combination of B6 and B7 insertion editing RECCs.

Different mutations of B6, B7 and B8 resulted in somewhat different PCR product profiles for each transcript, and the extent of the effects of certain mutations were also transcript specific. For example, BF B7 H125L mutation generally resulted in different effects on editing compared to other mutations in B7 (e.g. RAM) and significantly decreased A6 and ND7-5′ but not MURF2, RPS12, ND8 or CYb editing. These results likely reflect alterations in specific steps of editing performed by a particular RECC rather than a total loss of the RECC or its function. The profiles of the edited PCR products also differ between BF and PF life cycle stages in response to mutations, particularly in B7 and B8. For example, most mutations in B7 (e.g. ZnF, L291F, H313L, L325P and I330K) resulted in differential impacts to editing of A6, MURF2, RPS12, ND7-5′, ND8, and/or CYb in BF and PF. Additionally, several B8 mutations (e.g. ZnF, T175K, L204F, L319F and L368P) resulted in differential impacts to editing of A6 and RPS12 in BF versus PF, while others (R58C, G232R and I336F) differentially altered ND7-5′ editing. The relatively small A6 and RPS12 products in BF but not PF cells that exclusively express the WT B8 allele may be the consequence of B8 over-expression from the tubulin locus, which may result in increased abundance of N1/B8 RECC. Importantly, editing of the developmentally regulated CYb and ND8 transcripts did not correlate with life cycle stage-specific growth defects in e.g. B7 L291F, and B8 R58C, T175K, L204F, L319F and L368P. It therefore appears that stage-specific growth defects likely result from stage-specific editing defects at a subset of sites in multiple transcripts, rather than a general down-regulation of developmentally regulated mRNAs.

Taken together these RT-PCR data show that mutations across the three proteins result in various editing defects with (i) different mutations within the same protein or domain having distinct alterations in the editing of each transcript, (ii) single mutations resulting in distinct editing defects in different transcripts and (iii) life cycle-dependent differences in the editing of specific transcripts that indicate functional RECC differences between life cycle stages.

### Consequences of B6, B7 and B8 mutations to RECC associations

**Figure 6.**
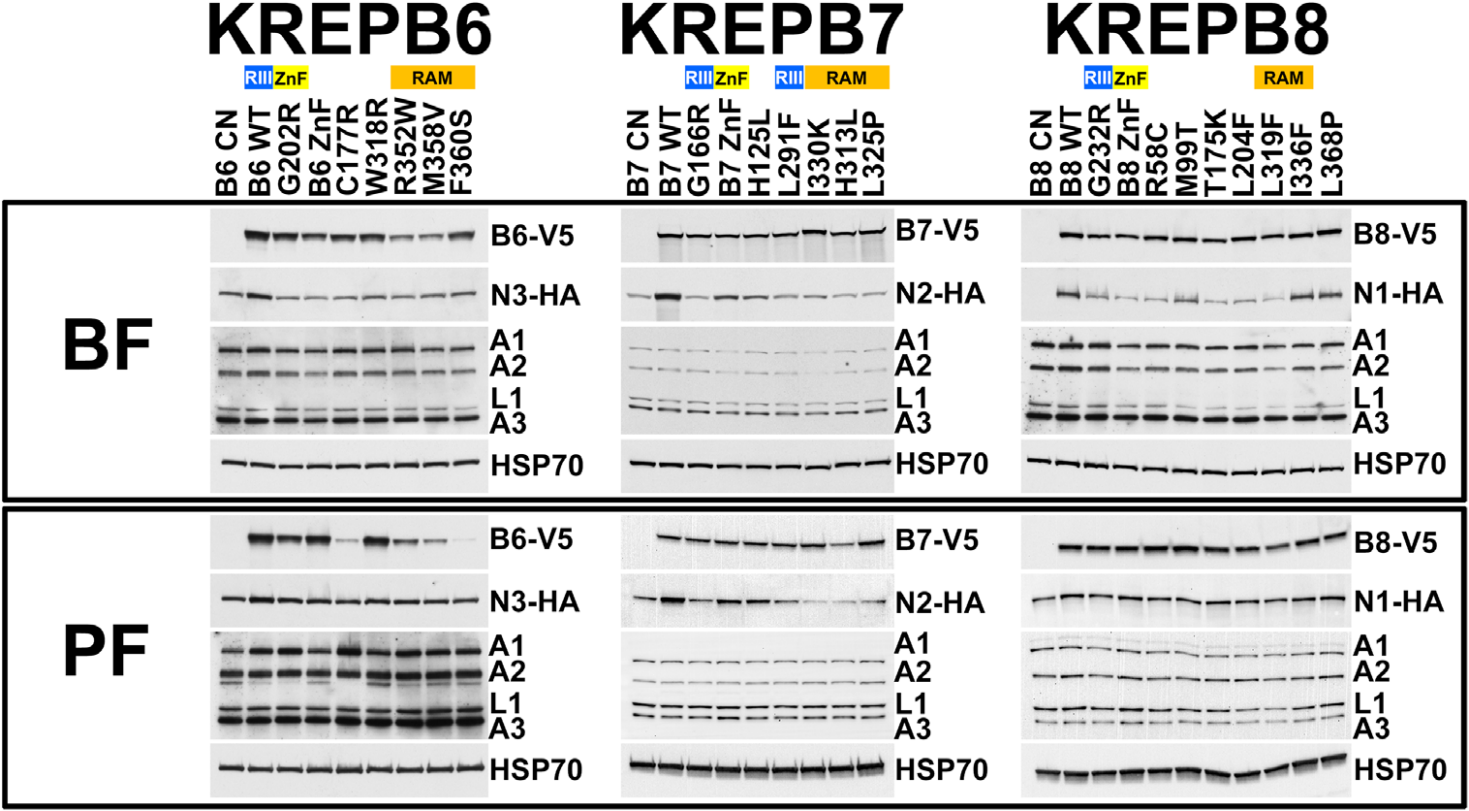
SDS-PAGE Western analyses of mutant cell lysates. Blots were probed with antibodies for V5 tagged B6, B7 or B8; HA tagged cognate endonuclease N3, N2, or N1, respectively; RECC proteins A1, A2, L1 and A3, and anti-HSP70 for mitochondrial heat-shock protein 70 as a loading control. See Figure 7 for BN-PAGE western analysis of the same lysates.

**Figure 7.**
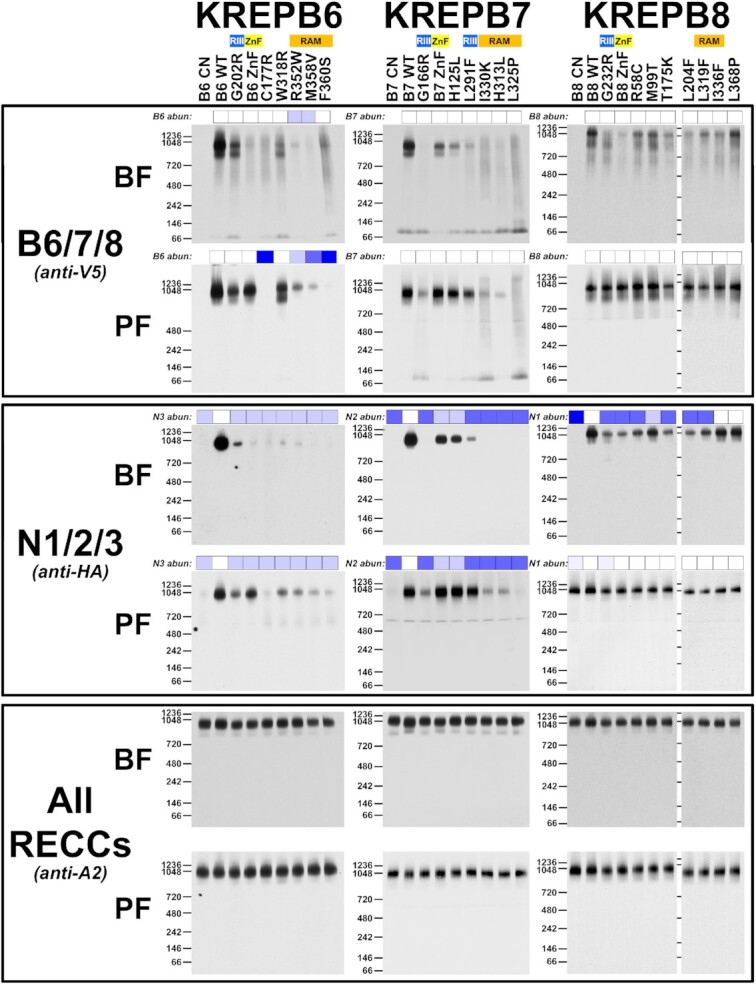
Consequences to RECC protein associations upon exclusive expression of mutant alleles. BN-PAGE and Western blots of cell lysates were probed using antibodies against V5 tagged B6, B7 or B8 (top panels); HA tagged cognate endonuclease N3, N2 or N1, respectively (middle panels); and the A2 core component of RECC (bottom panels). Small boxes above blots represent the loss of tagged B or N proteins in Figure 6 with darker blue colors indicating greater loss; see Figure 8 for additional information. RECC generally migrates at ∼1 MDa and some samples have a ∼800 kDa subcomplex.

We determined the consequences of the selected aa substitutions on protein abundances and RECC interactions in cells that exclusively expressed WT or mutant versions of B6, B7, or B8 either by SDS- (Figure 6) or blue native (BN)-PAGE (Figure 7) western analyses. Blots were probed for V5-tagged B6, B7 or B8, their respective HA-tagged cognate N3, N2 or N1 endonucleases, or four proteins that are common to RECCs (A1, A2, L1, and A3). Probing for A2 serves as an internal control in BN-PAGE, while HSP70 serves as a loading control for SDS-PAGE. We observed expression and associations of the B proteins and their tagged cognate endonucleases with ∼1 MDa RECCs in cells that exclusively express WT B6, B7 or B8. In contrast, exclusive expression of the selected B6, B7 or B8 mutant alleles had a variety of consequences to the abundances and associations with RECC of the B proteins and their cognate endonucleases, with some being lost, others disrupted and yet others seemingly unaffected (Figures 6 and 7). In addition, some mutations had effects that differed between life cycle stages. The relative abundances of four proteins (A1, A2, L1 and A3) that are common to all three RECCs remained similar in all cell lines tested, and all cells contained ∼1 MDa RECCs (see A2 probing of BN-PAGE). This is consistent with previous studies of RECC sedimentation upon loss of B6-B8 (15), and presumably reflects the presence of two unperturbed RECCs that remain after the knockdown or disruption of either B6, B7 or B8 specific RECCs.

*Effects of B6 mutations:* Exclusively expressed WT and mutant B6 proteins were present at comparable levels to each other in BF except for R352W and M358V, which were similarly less abundant in PF where C177R and F360S levels were also dramatically lower (Figure 6). WT B6 is predominantly associated with ∼1 MDa RECC in BF and PF, with a smaller proportion in a ∼800 kDa subcomplex in BF (Figure 7). The ∼800 kDa subcomplex is consistent with RECCs that lack the insertion heterotrimeric subcomplex (KRET2–KREPA1–KREL2) (20), and its presence could be due to overexpression from the tubulin locus and subsequent alteration of RECC stoichiometries in these cells. This subcomplex is also observed in mutants, such as W318R, in which it is more prominent in PF. Multiple mutants also had bands consistent with B6 that was not incorporated in larger complexes that were observed near the bottom of these native gels. The associations of R352W and M358V mutant proteins with complexes and/or subcomplexes reflected their abundances in BF. However, several B6 mutants (e.g. G202R, ZnF, C177R, W318R and F360S) were reduced in complexes and subcomplexes in BF despite their total abundances being somewhat similar to WT. In contrast, the presence of B6 mutants in PF RECCs consistently mirrored their abundances.

N3 cognate endonuclease abundance was slightly reduced in all BF B6 mutants relative to WT, but N3 association with RECCs was substantially reduced in all BF mutant cell lines (Figures 6 and 7), generally, but not always (e.g. F360S) correlating with the RECC associations of the mutant B6 proteins themselves. In PF, reduction in N3 abundance relative to WT was more moderate than in BF for most mutants, and N3 association with RECCs consistently mirrored the abundances and RECC associations of the B6 mutants (Figures 6 and 7). Thus, the association of N3 with RECC correlated with its cellular abundance and/or B6 association with RECC in both BF and PF. Life cycle stage differences in RECCs were observed in differential presence of B6 and N3 as was especially evident in the BF vs. PF ZnF mutants.

*Effects of B7 mutations:* Analysis of B7 mutants also showed distinct B7 and N2 abundance and RECC association phenotypes. Exclusively expressed WT and mutant B7 proteins were mostly present at similar levels to each other in both BF and PF, except H313L which was reduced compared to WT in PF (Figure 6). WT B7 predominantly associated with ∼1 MDa RECC, with a smaller proportion found in a ∼800 kDa subcomplex in BF as with B6 (Figure 7). Most mutant B7 proteins were not primarily associated with ∼1 MDa RECCs in BF except for ZnF and H125L mutants, although their RECC association was also reduced, and the ZnF mutant was associated with a subcomplex. As in BF, the H313L, L325P, and I330K RAM motif mutants did not associate with RECC in PF. In contrast, RECC association of the G166R mutant was reduced but detectable in PF, while the ZnF, H125L and L291F mutants behaved similarly to WT.

N2 levels in BF correlated with the association of mutant B7 with RECC, with little effect in ZnF and H125L mutants, reductions in G166R and L291F mutants and large reductions in H313L, L325P and I330K mutants (Figure 6). These mutations had similar, albeit smaller effects on N2 levels in PF. RECC-associated N2 was not detectable in the G166R, H313L, L325P, I330K mutants in BF, and was reduced in PF. ZnF, H125L and L291F mutants resulted in reduced N2 associations with RECC in BF but not PF (Figure 7). Thus, the abundance and RECC association of N2 correlated with the RECC association of mutant B7 in both BF and PF.

*Effects of B8 mutations:* Mutations in B8 had little effect on B8 protein levels (Figure 6). WT B8 was associated with both ∼1 MDa RECC and ∼800 kDa subcomplex in BF, but all the mutant proteins had reduced associations with RECC to varying degrees, particularly G232R, ZnF, T175K, L204F, L319F and I336F (Figure 7). In contrast, all mutant proteins were associated with RECCs at WT levels in PF, underscoring life cycle stage differences in RECC protein associations (Figure 7), perhaps involving the X1 exonuclease that is uniquely found in B8/N1 RECCs and crosslinks with both N1 and B8 (22). Several mutations resulted in reduced levels of N1 in BF but not PF, and the BF reduction in N1 abundance correlated with B8 mutant RECC association. The association of N1 was almost exclusively with ∼1 MDa RECCs and was reduced to varying degrees in the BF mutants, reflecting the abundance of N1 in the cells. Mutant I336F is notable because N1 levels and RECC incorporation were similar to those upon exclusive expression of the WT allele in BF, despite reduced association of B8.

**Figure 8.**
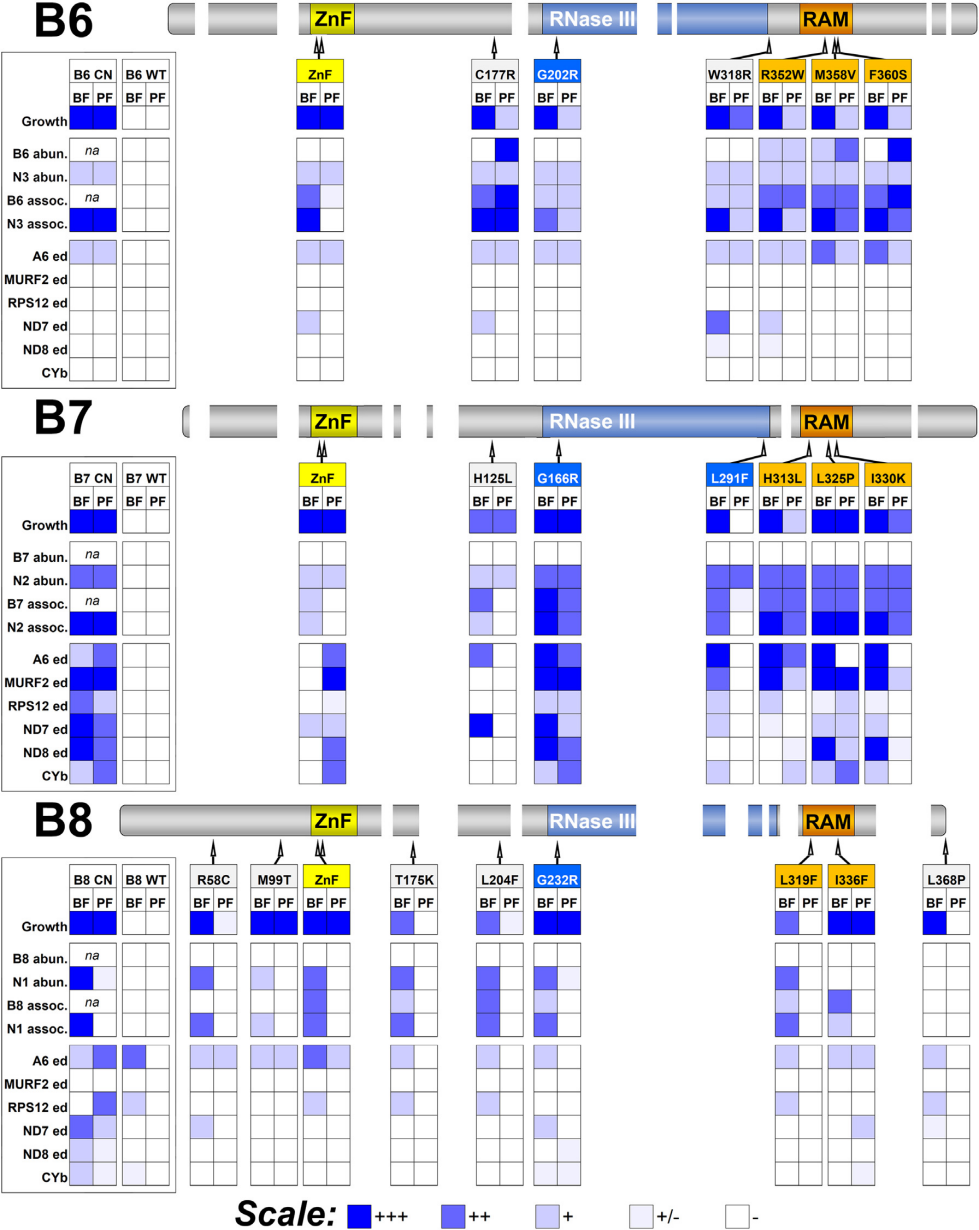
Summary of phenotypes resulting from exclusive expression of mutant B6, B7, or B8 alleles. The magnitude of impacts on growth, B6/7/8 and cognate N1/2/3 protein abundances and their associations with RECC, as well as editing of A6, MURF2, RPS12, ND7-5′, ND8, and CYb mRNAs are indicated using a heat map which indicates large (+++), medium (++), small (+), marginal (+/−), or no impact (−); na = not applicable. Locations of mutations are indicated in B6/7/8 schematic alignment from Figure 3. See Figures 4–7 for primary data. Zinc Finger (ZnF) and RNase III motifs and the RNase III Associated Motif (RAM) are indicated.

Overall, the results from denaturing and BN-PAGE western analyses of mutant proteins show a variety of effects on B/N abundances and associations with RECC among B6-B8, some of which differ between life cycle stages (Figure 8). Furthermore, despite their broad similarities, mutation of these proteins results in distinct consequences even when homologous residues are changed, illustrating the distinct characteristics of the three RECCs that function together to perform editing. For example, the G202R mutation of B6 and the corresponding G166R mutation of B7 had different effects, as did ZnF mutations in these two proteins. B7 G166R did not associate with RECC in BF and its association was much reduced in PF, with parallel reductions in N2 levels and RECC association. In contrast, B6 G202R associated with RECC in both BF and PF, albeit to a reduced level compared to WT, but also resulted in reductions in N3 levels and RECC association. Similarly, B7 ZnF associates with RECC in both BF and PF, but B6 ZnF associates with RECC in PF but not BF. That mutations at comparable positions have different consequences implies that B6-B8 interact somewhat differently with other (common) RECC proteins which may contribute to functional differences.

### Mapping substitutions onto predicted B6, B7, and B8 structures

**Figure 9.**
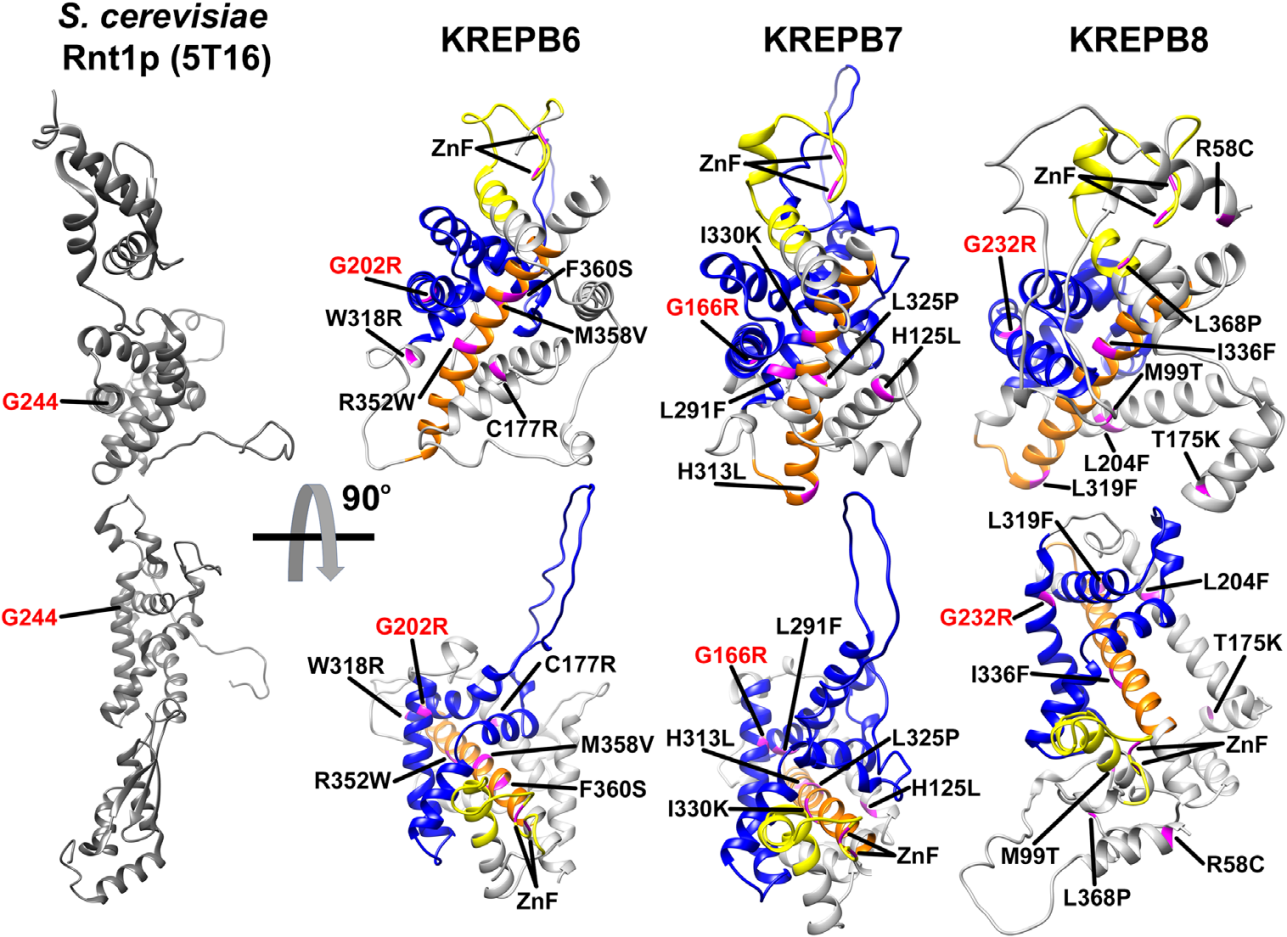
Predicted Alphafold structures of *T. cruzi* B6, B7 and B8 compared to the crystal structure of *S. cerevisiae* Rnt1p RNase III monomer. The lower structures are rotated by 90° toward the viewer with the ZnF motif in yellow, RNase III motif in blue, and RAM in orange. The functionally important amino acids that were characterized in both BF and PF *T. brucei* are highlighted in magenta, and the universally conserved glycine in the RNase III signature motif is highlighted in red in all structures.

**Figure 10.**
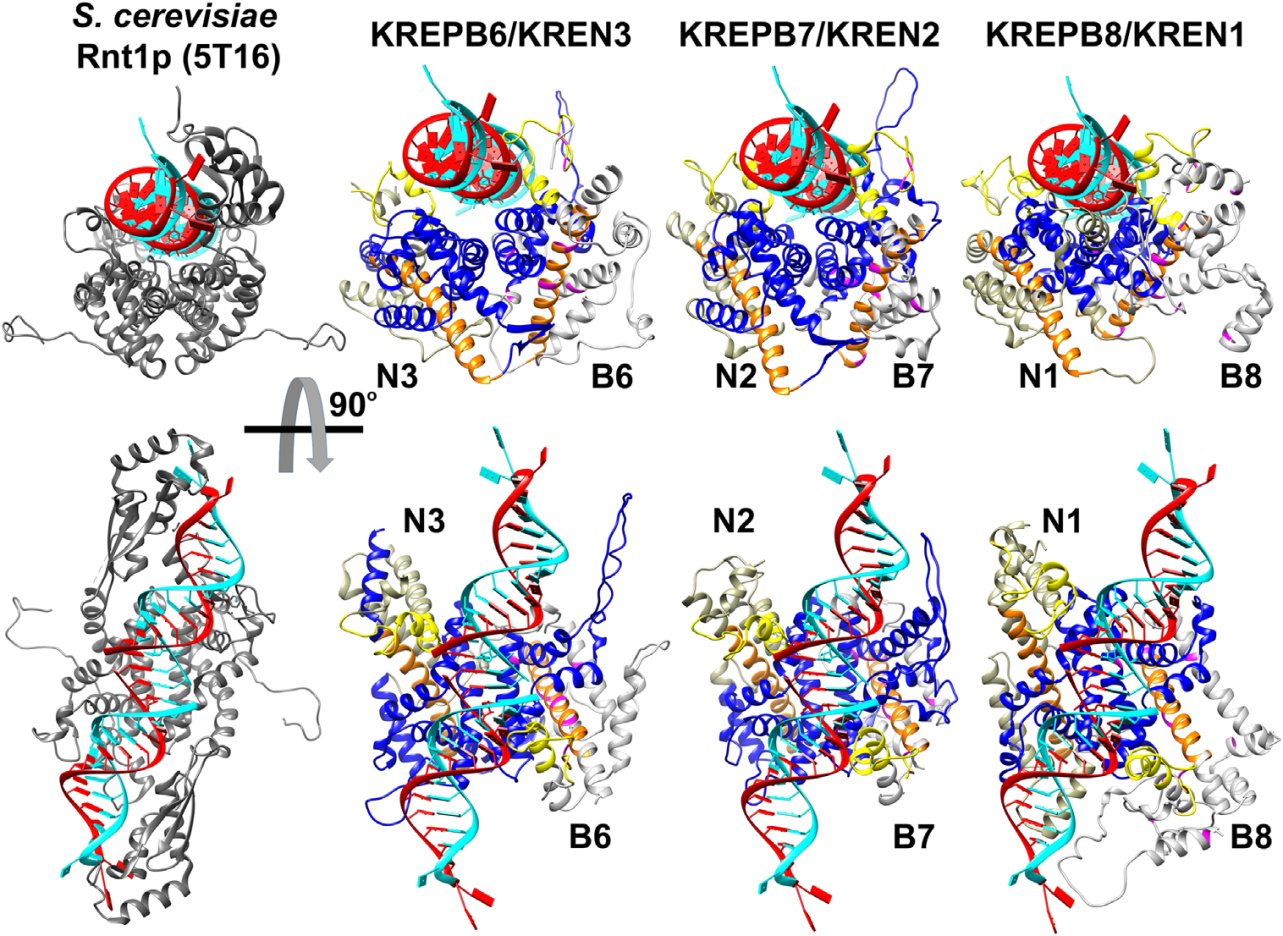
Models of B6/N3, B7/N2, and B8/N1 dimers with RNA based on the predicted *T. cruzi* B6-B8 and N1-N3 Alphafold structures, and the crystal structure of the *S. cerevisiae* Rnt1p RNase III dimer with RNA substrate (PDB structure 5T16). The lower structures are rotated 90° toward the viewer and the RECC ZnF motif is yellow, the RNase III motif is blue, and RAM is orange. Locations of functionally important amino acids in both BF and PF are colored magenta. Dimeric Rnt1p RNase III domains cleave both RNA strands but only the N partner of the B6/N3, B7/N2 and B8/N1 heterodimers has a catalytic amino acid that is positioned for cleavage of the red RNA strand. The catalytically non-functional B partner cannot cleave the cyan RNA strand. Note that the placements of ZnF motifs in RECC structures mirror the positions of the dsRBDs of Rnt1p that bind substrate RNA.

The recent release of predicted *T. cruzi* structures for all full-length RECC RNase III paralogs by Alphafold (42) provides a powerful resource to understand the effects of our aa substitutions on B6-B8 function. We utilized the predicted structures of the *T. cruzi* B6-B8 orthologs as they share high sequence identities with the *T. brucei* proteins (52.2%, 81.7% and 75.0%, respectively) (Supplementary Figures S4–S6). For clarity we removed low confidence and disordered regions at the N and C-termini of each of the proteins that were unstructured (Supplementary Figures S4–S6). Comparison with the crystal structures of archetypal eukaryotic (yeast Rnt1p; PDB 5T16) and bacterial (RNase III; PDB 2NUF) RNase III proteins confirmed the presence of RNase III motif-like folds in the predicted B6, B7, and B8 structures (Figure 9 and Supplementary Figure S7) as expected from our previous homology modelling of the B6-B8 RNase III motifs (22,47,48). Here we modelled the predicted structures for B6, B7, and B8 and their cognate N1, N2, and N3 endonucleases (Supplementary Figures S8–S10) onto these archetypal RNase III crystal structures and built dsRNA substrate-containing models for the putative B6/N3, B7/N2, and B8/N1 dimers that form the proposed active sites that cleave mRNAs at ESs (Figure 10 and Supplementary Figure S7). The amino acids that coordinate Mg^2+^ ions in the catalytic active sites in N1, N3 and N3 are precisely aligned with homologous residues in both the eukaryotic and bacterial RNase IIIs in these models (Supplementary Figure S7B), providing strong validation for the predicted structures and our models.

We further validated the predicted structures and our dimer models using our previous BS3 crosslinking-mass spectrometry data (22). BS3 has a linker arm of 11.4 Å when fully extended and can crosslink two residues whose Cα atoms are up to 30 Å apart (49). We measured the distances between the residues in the *T. cruzi* orthologs that aligned with the intra- and inter-linked residues in the *T. brucei* B6-B8 and cognate N1 - N3 endonucleases and that could be mapped onto the predicted structures. We were able to measure the distances for intralinks in all proteins and one interlink between B6 and N3. In most cases the distances between crosslinked residues were <30 Å, showing that the predicted structures and models are consistent with the available experimental data and thus provide reasonable representations of the structures and interactions of B6/N3, B7/N2, and B8/N1 (Supplementary Figure S11). While crosslinking data for B6, B7 and B8 is limited, we also mapped residues in N1, N2, and N3 that crosslinked to other RECC proteins onto our models where possible, including proteins that crosslinked to all three endonucleases (B4, A1, T2), and the catalysts for the editing steps following substrate cleavage (T2 and X1). These analyses revealed that RECC proteins B4, A1 and T2 are (i) in proximity to residues that are on different surfaces than the partner B6–B8 protein interfaces, thereby providing further confidence in our models and (ii) in proximity to equivalent positions in the N1–N3 endonuclease structures, indicating that each of the endonucleases interacts somewhat similarly with these other RECC proteins (Supplementary Figure S12). Interestingly, endonuclease crosslinks to the T2 and X1 enzymes are found near the cleavage site in the RNA substrate in our models, suggesting close coordination between cleavage and U addition or deletion.

These structures and our models refine and extend the previously annotated RNase III motifs within the B proteins and their cognate endonucleases and provide the most detailed views to date of the active sites for mRNA cleavage in RECC (Figure 10 and Supplementary Figure S7). Mapping of the single amino acid substitutions that disrupted B6, B7 or B8 function onto the models (Figures 9 and 10) also provides structural context for understanding their phenotypes. Notably, the newly identified RAM is part of an alpha helix present in all predicted RECC RNase III paralog structures, where it forms a backbone running through the RNase III motifs upon which other helices can pack and potentially providing a binding surface for other RECC proteins. Accordingly, the RAM mutations in B6, B7, and B8 result in strong B protein and cognate endonuclease RECC association defects that are consistent with protein-protein interaction and RNase III motif stabilizing roles for this motif. Strikingly, the models also suggest gRNA/mRNA heteroduplex binding roles for the ZnF motifs within both the B proteins and their cognate endonucleases, placing them either side of the RNA substrate near the cleavage site. The ZnF motifs in these proteins therefore appear to play equivalent roles to the dsRBD domains that are present in the classical RNase III proteins (Figure 10 and Supplementary Figure S7).

## DISCUSSION

Random mutagenesis and complementation screening, which we adapted for *T. brucei* (16), enabled us to identify multiple essential functional motifs in B6, B7, and B8 RECC proteins from a total of 43 single aa mutations. These mutations had various consequences in different life cycle stages on parasite growth, on the abundances and associations of these proteins and their cognate endonucleases with RECCs, and on RNA editing (summarized in Figure 8). These consequences indicate (i) the specific functions in editing of each domain of these three proteins and (ii) the complementary functions of the three RECCs that are essential and perform numerous catalytic cycles of editing that collectively result in the functional maturation of multiple mt mRNAs.

*RNase III:* Mutations in the RNase III motifs of B6, B7, and B8 had consequences that reinforce our earlier conclusions that B6/N3, B7/N2 and B8/N1 function as three related RNase III heterodimers in the three different RECCs (15,20). This is exemplified by mutations of highly conserved G (G202R, G166R, G232R) and R (R310A, R288A, R301A) residues in the RNase III motifs of B6, B7 and B8 respectively which resulted in severe growth defects and decreased abundances and RECC associations of N3, N2, and N1 (15). Other mutations in the RNase III domain of these three proteins were similarly disruptive, including B7 L291F and B8 L204F which also impacted N2 and N1 abundance and RECC association respectively, at least in BF. Overall, these results indicate that the B6, B7 and B8 RNase III motifs are required for interactions with the cognate N proteins and with RECCs and that they are essential for the selective cleavage of mRNA ESs and hence accurate editing.

*RAM:* The multiple mutations in the novel RAM regions of B6, B7, and B8 led to defects in growth, editing, and RECCs indicating that this motif broadly contributes to RECC function, and RNase III structural integrity by the association of the B proteins with RECCs and the stability of the cognate N proteins. The structural predictions of B6-B8 and N1-N3 RAMs indicate that they form similar elongated alpha-helical structures that flank and associate with the RNase III motifs, underscoring their relationship with RECC catalytic function and structure (Figures 9 and 10). The RAM helix thus appears to contribute to the structural framework in RECC that organizes the active site of each heterodimeric RNase III endonuclease. Comparison of the equivalent B6-B8 and N1-N3 RAM alpha helices with those within yeast Rnt1p or bacterial RNase III’s indicate that those of B6-B8 are longer and curve in opposite directions relative to the ZnFs or dsRBDs, respectively. The bacterial RNase III helix directly interacts with Ymdb protein which negatively regulates RNase III activity (50). Differences between B6–B8 RAM and bacterial or yeast RNase III structures may therefore reflect differences in protein-protein interactions in addition to the lack of dsRBDs in B6–B8. Additionally, the RAM sequences are located where potential PUF motifs were noted in B4, B5 and all three endonucleases (15,22,45) (Supplementary Figure S3) and structural predictions indicate that this region in B4 and B5 can form elongated alpha helixes analogous to those predicted for B6-B8 and N1–N3 RAMs. These results therefore suggest that the PUF motif previously identified in B4, B5 and N1–N3 is instead a RAM involved in protein-protein interactions rather than with RNA, although the predicted structures are static minimal energy models that do not account for dynamic conformational changes that may occur during editing. Together, these data refine the annotated RNase III motifs in B6–B8, and N1–N3 by the addition of a novel RAM. In addition, they further highlight the difficulties in identifying divergent protein motifs in kinetoplastids using sequence analysis, further demonstrating the power of random mutagenesis and structural approaches in understanding protein function.

*ZnFs*: Mutation of the B6, B7 and B8 ZnFs indicate that they participate in the association of these proteins, their cognate endonucleases and/or other proteins and RNA substrates with RECCs. Our structural models of the B6/N3, B7/N2, B8/N1 heterodimers (Figure 10) place the ZnF of each cognate protein in contact with and on opposite sides of double stranded RNA at a position near the RNase III active site. These structures indicate that the B/N ZnFs play roles in binding and positioning RNA substrates similar to the dsRBD domains of eukaryotic and bacterial RNase IIIs (28). The B/N ZnFs belong to the matrin-type family of C2H2 ZnFs that have the C-x2-C-x-H-x5-H pattern, are in several RNA binding proteins including Matrin-3 and U1 small ribonucleoprotein C (U1C) (51–54), and are involved in direct RNA- and protein-protein interactions (53,55). Intriguingly, the ZnF of the U1C protein within the spliceosome snRNP component interacts with pre-mRNA/U1-snRNA duplexes at an early stage in RNA splicing and functions in the fine-tuning and stabilization of mismatched base-pairing within the duplexes (55). The U1C protein contacts with the RNA duplex involve hydrogen and electrostatic interactions with basic amino acids that are between the ZnF histidines, i.e. are not base-specific, and splice-site determination is governed by the thermodynamic stability of the pre-mRNA–snRNA duplex (53–55). Like the U1C ZnF, our models place basic amino acids in the B6/N3, B7/N2, B8/N1 protein ZnFs next to RNA, suggesting that these different motifs bind RNA heteroduplexes analogously. Furthermore, there are subtle differences between the B6/N3, B7/N2, B8/N1 ZnFs which might affect their binding to the different substrates and hence contribute to the different ES specificities of the three RECCs. For example, the spacing between the histidine residues in the N1 ZnF uniquely deviates from the H-x5-H matrin-type ZnF consensus of other RECC RNase IIIs, which may accommodate deletion vs insertion substrates.

The analysis above leads us to hypothesize that the ZnF, RNase III, and RAM structures act in concert in the positioning of the heteroduplex mRNA/gRNA substrate, with the mRNA ES proximal to a heterodimeric RNase III active site of B6/N3, B7/N2, or B8/N1. We propose that dimerization is mediated via the RNase III motif, and that the two ZnFs of the heterodimer govern RNA substrate base-pairing and generate various RNA conformers, perhaps dynamically, which differ in their potential as suitable substrates. By analogy with YmdB, substrate recognition and efficiency of cleavage is then influenced by the RNase III and RAM motifs within the overall RECC context. Mutations in each of these three domains would thus be predicted to have different effects on RECCs and editing that are consistent with our results. ZnF mutations would be predicted to affect substrate recognition and binding, those in RAM would affect editing efficiency and specificity, and those in RNase III would affect dimerization and cleavage. In addition, as we observe, some mutations in all three motifs would affect association of the B6/N3, B7/N2, or B8/N1 with RECCs depending on protein interactions within these complexes. Because loss of one N1, N2, or N3 affects the abundances of the remaining two endonucleases (20,23) it would also alter the stoichiometric balance of the three RECCs, contributing to the observed editing phenotypes. However, the interactions of the B6/N3, B7/N2 and B8/N1 ZnF, RNase III, and RAM motifs alone are unlikely to be sufficient for interaction and positioning of the sizeable gRNA/mRNA heteroduplex substrates within RECCs during editing. Furthermore, editing of each ES requires three catalytic steps of mRNA cleavage, U insertion or deletion, and RNA ligation, which must entail a series of dynamic interactions between the RNA and these three domains of the B/N endonuclease and domains of other RECC proteins, including the catalysts (4) (Supplementary Figure S12). The editing of mRNA blocks where a single gRNA specifies multiple insertion and deletion ESs requires the successive action of the B6/N3, B7/N2 and B8/N1 RECCs which have different ES specificities (19,21,23,24). Hence, the editing of a mRNA block specified by such a gRNA cannot be accomplished by a single RECC but the coordinated actions of multiple RECCs and perhaps other proteins and complexes, e.g. RESCs or REH2C (1,4).

*Other paralogs:* Four other RECC associated proteins, B4, B5, B9 and B10, are paralogous to the heterodimeric B/N endonuclease proteins, each containing a matrin-type ZnF, an RNase III motif that is non-catalytic, and a RAM (Supplementary Table S1). Their predicted structures are very similar to those of B6-B8 and N1-N3 (Supplementary Figure S13) (42). B4 and B5 are present in all three RECCs and are essential for editing. Knockdown and mutational studies have shown that unlike B6-B8, B4 and B5 are required for the gross maintenance of RECC integrity, perhaps via a broader network of protein-protein interactions within RECC as suggested by crosslinking studies (22). B9 and B10 are more closely related to B8 and each other than to the other seven paralogs. They are not essential for editing nor consistently stably associated with the three RECCs at least in the stages and under the conditions tested (37). Thus, their association with RECCs may be transient or with minor RECC populations. Mutational analyses, including of the highly conserved RNase III glycine residues, revealed that as seen with B6-B8, the B4, B5, B9, and B10 RNase III motifs participate to varying degrees in the associations of these proteins with RECCs (14,16,37,56). Interestingly, knockdown or RNase III mutation of B4 also resulted in reduced abundances and RECC associations of N1-N3. This, and the proximities between B4–B8 and B10 and the N1–N3, led us to suggest that other RNase III-mediated heterodimerizations might occur between different pairs of these proteins, affecting RECC interactions with the numerous substrates at different steps during editing (15). In addition, by analogy with the B/N structural models, the B4, B5, B9 and B10 ZnFs may also function in substrate positioning. Intriguingly, the B5 ZnF, which deviates from a canonical matrin-type ZnF sequence (56), is not essential for function, suggesting that it may not interact or may be interacting differently with RNA substrate and/or other RECC proteins than other B and N ZnFs.

*Life-cycle stage differences*: The mutations we identified in B6-B8 resulted in a range of growth effects in PF versus BF. Some mutations affected growth, editing, and RECCs in both BF and PF, others had growth defects in both stages but differential effects on editing, protein abundances and/or RECCs, and yet others had these effects in BF, where screening was done, but not in PF. Mutations in the RNase III and RAM regions severely disrupted growth, editing, and RECCs in both BFs and PFs (Figure 8). These include the RNase III G202R, and G166R mutations in B6 and B7, that reduce the abundances and disrupt RECC association of their cognate endonucleases (15). The consequences of the substitutions to these highly conserved RNase III glycine residues are consistent with our hypothesis that RNase III domain dimerization is essential for endonuclease stability and function in both life cycle stages. Similarly, all mutations in the B6 and B7 RAM also disrupted PF growth, editing, and the abundances and association with RECC of B6, B7 and their cognate endonucleases. The impacts of RAM mutations in both life cycle stages are a further indication that these motifs are a major contributor to the structural framework of the RNase III active site that is essential for endonuclease function in both BF and PF.

Interestingly, all B8 mutations that strongly disrupted growth in both BF and PF, including in the RAM and of the highly conserved RNase III glycine residue, affected cognate N1 endonuclease abundance and RECC association in BF only. We hypothesize that N1 may persist in PF due to additional, potentially stage-specific interactions with X1 or B10, both of which have been shown to crosslink with N1 (22). While the precise function of B10 is not clear, it appears to have arisen from a gene duplication of B8 and thus these proteins may have some functional similarities that include association with N1 (22,37). Some B6 and B7 mutations also resulted in growth and editing defects in both BF and PF but had differential effects on editing and/or RECCs between the two life-cycle stages (Figure 8). For example, the ZnF motifs in B6, B7 and B8 are essential in both life cycle stages, but there were striking differences between these stages in RECC association and profiles of pre-, partially-, and fully edited mRNAs. The association of the ZnF mutant B6-B8 proteins with RECCs and the abundances of their cognate endonucleases were primarily disrupted in BF versus PF. Therefore, growth and editing defects correlated with absence of the B and N proteins in RECC in BF, but these defects occurred in PF despite the presence of these proteins in RECC. Thus, mutations can result in growth and editing defects in different ways in BF versus PF, e.g. altered protein-protein interactions and endonuclease stoichiometry in BF versus altered recognition of substrates by the B protein and/or endonuclease in PF.

Strikingly, we also identified B6, B7, and B8 mutations that had little or no apparent deleterious effects on PF growth, editing and RECCs (Figure 8). These include mutations in the RNase III (e.g. B7 L291F and B8 L204F) and RAM (e.g. L319F) regions, in addition to mutations outside of the ZnF, RNase III, and RAM (e.g. B8 T175K and L368P). Many of these mutations are further from the RNA substrate or RNase III active site in our structural predictions than those with strong defects in both BF and PF. These results suggest that the structure of the RNase III active site required for ES cleavage is similar between BF and PF, but other regions of B6-B8, including within the RNase III and RAM function and/or interact with RECC (and/or other proteins) differentially between BF and PF.

Life-cycle stage-specific effects on growth and editing were also previously observed for a range of single amino acid substitutions in other RECC proteins, most prominently B5 (14,16,17), but with some fundamental differences in comparison to the observed B6, B7 and B8 phenotypes. B5 mutants that resulted in growth defects also significantly disrupted protein abundances and integrities within all RECCs (16). In contrast, multiple B6-B8 mutants with growth defects had minimal impacts on RECC protein abundances and associations (e.g. BF B8 L368P, PF B6-B8 ZnF and PF B8 G232R and I336F). The results of RT-PCR profiling of edited transcripts in the B6-B8 mutants also reflect more subtle functional defects than those observed in B5 mutants, as almost all B6-B8 mutants examined had transcript-specific defects in addition to life-cycle stage-specific effects. Interestingly, life-cycle stage-specific defects in the growth of B6-B8 mutants did not correlate with defects in editing of the developmentally regulated transcripts ND8 and CYb, but with defects in the other transcripts assayed that are usually edited in both life-cycle stages. We therefore hypothesize that life-cycle stage-specific defects in the growth of B6-B8 mutants result from editing defects at a subset of sites in a specific stage rather than a targeted impact on developmentally regulated mRNAs, or a wide-ranging loss of editing in a specific stage like that previously observed for B5 mutants. Thus, life-cycle stage-specific effects on growth and editing observed in both B5 and B6–B8 random mutagenesis experiments appear to arise via different mechanisms due to the broad functional differences between B5 and B6-B8.

One similarity that the previous B5 and the current B6-B8 studies share is that the consequences of most mutations on RECCs are generally more severe in BF than in PF. The differential stabilities and/or functions of BF and PF RECCs and RECC proteins upon B5, or B6, B7 and B8 knockdown or mutation indicate that BF RECCs and RECC components are generally more sensitive to perturbation (14,16,17). The molecular basis for this does not appear to involve differences in protein composition (20). Instead, our work collectively implies that there are numerous structural and functional differences in multiple RECC proteins between BF and PF RECCs. These are contemporaneous with the extensive changes in nuclear gene expression that occur during the trypanosome life cycle that are responses to external and internal stimuli, e.g. temperature, surface protein expression, metabolites, and other soluble factors (57,58). The precise differences between BF and PF RECCs reflected in our data could include RECC protein post-translational modifications and conformations, and their molecular interactions within RECCs, with substrate RNAs, and with other complexes e.g. RESC and REH2C. These characteristics would be expected to impact specific functions such as the rates and specificities by which ESs are edited between the two life cycle stages, thus resulting in developmental regulation of editing.

Overall, this study has (i) refined motif annotations within all RECC endonuclease paralogs via sequence analyses and structural modelling, (ii) provided a structural context for understanding the collaborative roles of the ZnF, RNase III and RAM regions within them and (iii) highlighted functional differences between them, including between life cycle stages, via comparisons of mutant phenotypes. Detailed biochemical and structural analyses are required to determine the nature of heteromeric RECC endonuclease paralog interactions, and of their interactions with RNA substrates and other RECC proteins in different *T. brucei* life cycle stages. RNase III-mediated RNA cleavage, RNA binding by ZnFs, and coordination between multiple RNA and protein binding motifs are crucial to the regulation of gene expression across eukaryotes, bacteria, and viruses. However, the presence of multiple potential RNase III heterodimers with both catalytic and non-catalytic components so far seems to be unique to kinetoplastid RECCs. Thus, understanding the detailed molecular mechanisms of editing and its regulation may reveal new therapeutics for treating kinetoplastid diseases. Such insights would also provide new paradigms for understanding the fundamental and collaborative roles of endonuclease domains in the regulation of gene expression.

## DATA AVAILABILITY

Models for B6/N3, B7/N2, and B8/N1 heterodimers were generated by superposition of the Alphafold predicted structures for the *T. cruzi* orthologs onto PDB structures 5T16 and 2NUF using the Chimera Matchmaker tool. Model coordinates and parameters are provided as supplementary information. The models were validated using our previous BS3 crosslinking-mass spectrometry data (Supplementary Figure S11) (22).

## Supplementary Material

gkac753_Supplemental_Files
