# Supplementary material for: Domain function and predicted structure of three heterodimeric endonuclease subunits of RNA editing catalytic complexes in Trypanosoma brucei
Source: Nucleic Acids Res. 2022 Sep 12;50(17):10123–39. doi: 10.1093/nar/gkac753 (PMC9508840; doi:10.1093/nar/gkac753)
Supplement: gkac753_Supplemental_Files [file gkac753_supplemental_files.zip › B678_NAR_Supplementary_Information_Revised_Final.pdf]

Supplementary Figure 1.

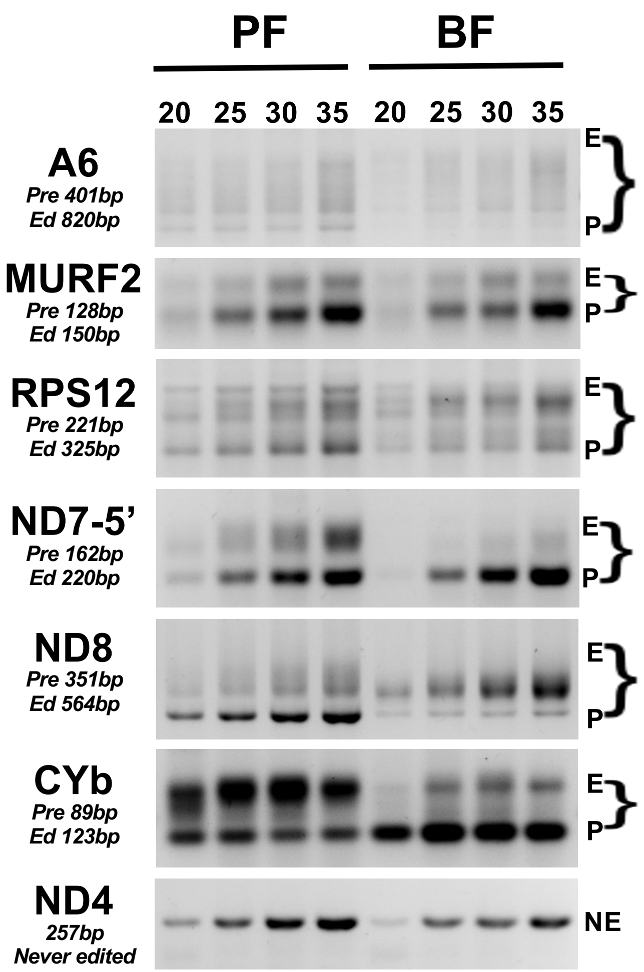

Supplementary Figure 2.

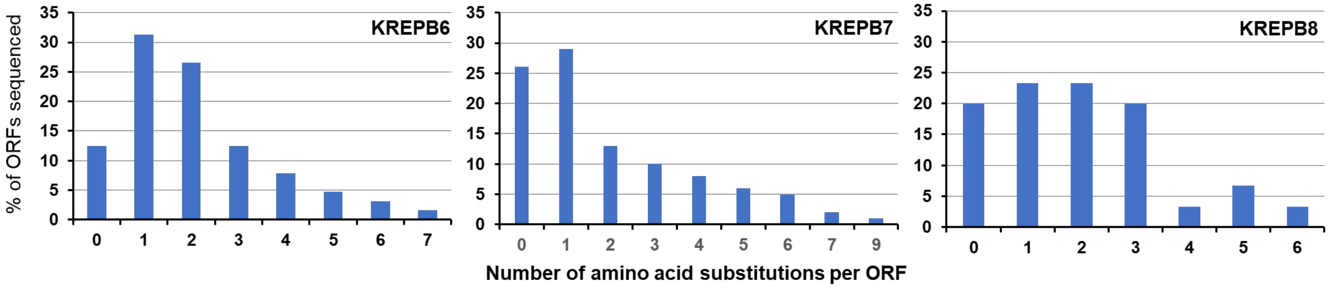

Supplementary Figure 3.

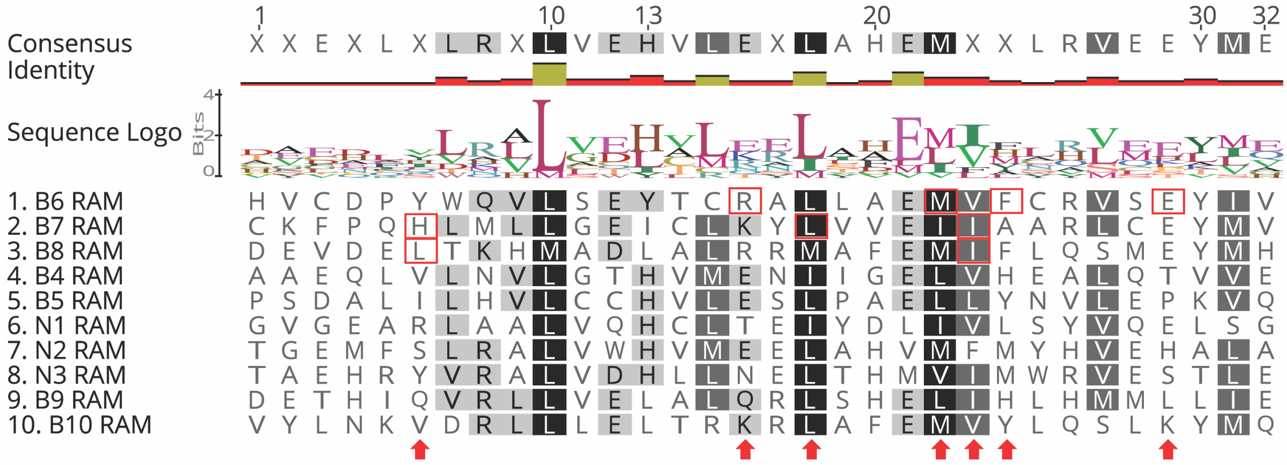

Supplementary Figure 4.

T cruzi KREPB6 (Q4CPS4) Alphafold model
Deleted low and very low confidence regions at N and C termini (AlphaFold pLDDT score < 70) M1-S107 and G416-N475*
Deleted regions correspond *to T. brucei* M1-T59 and G378-R438

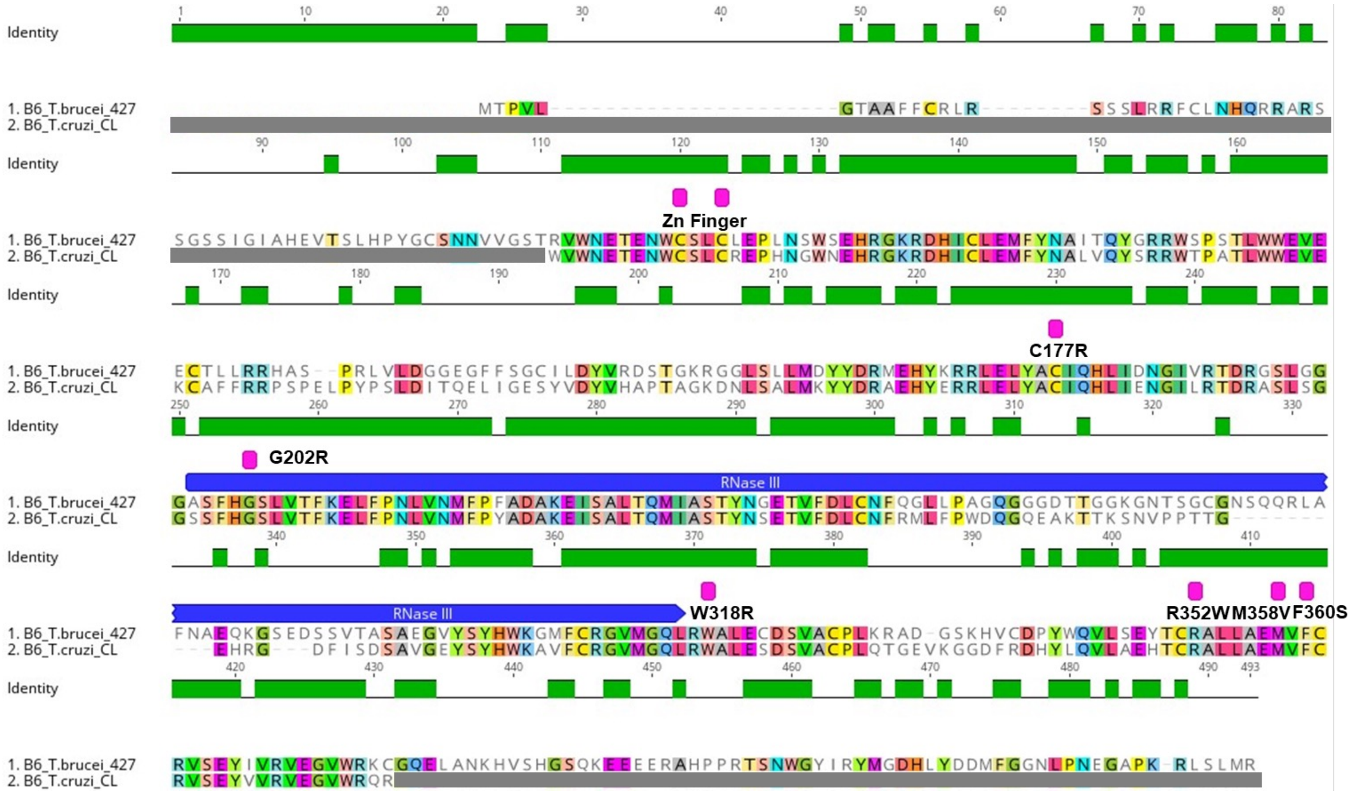

Supplementary Figure 5.

T cruzi KREPB7(Q4DHG3) Alphafold model
Deleted low and very low confidence regions at N and C termini (AlphaFold pLDDT score < 70) M1-P54 and P346-A414*
Deleted regions correspond to *T. brucei* M1-P55 and P343-V411*

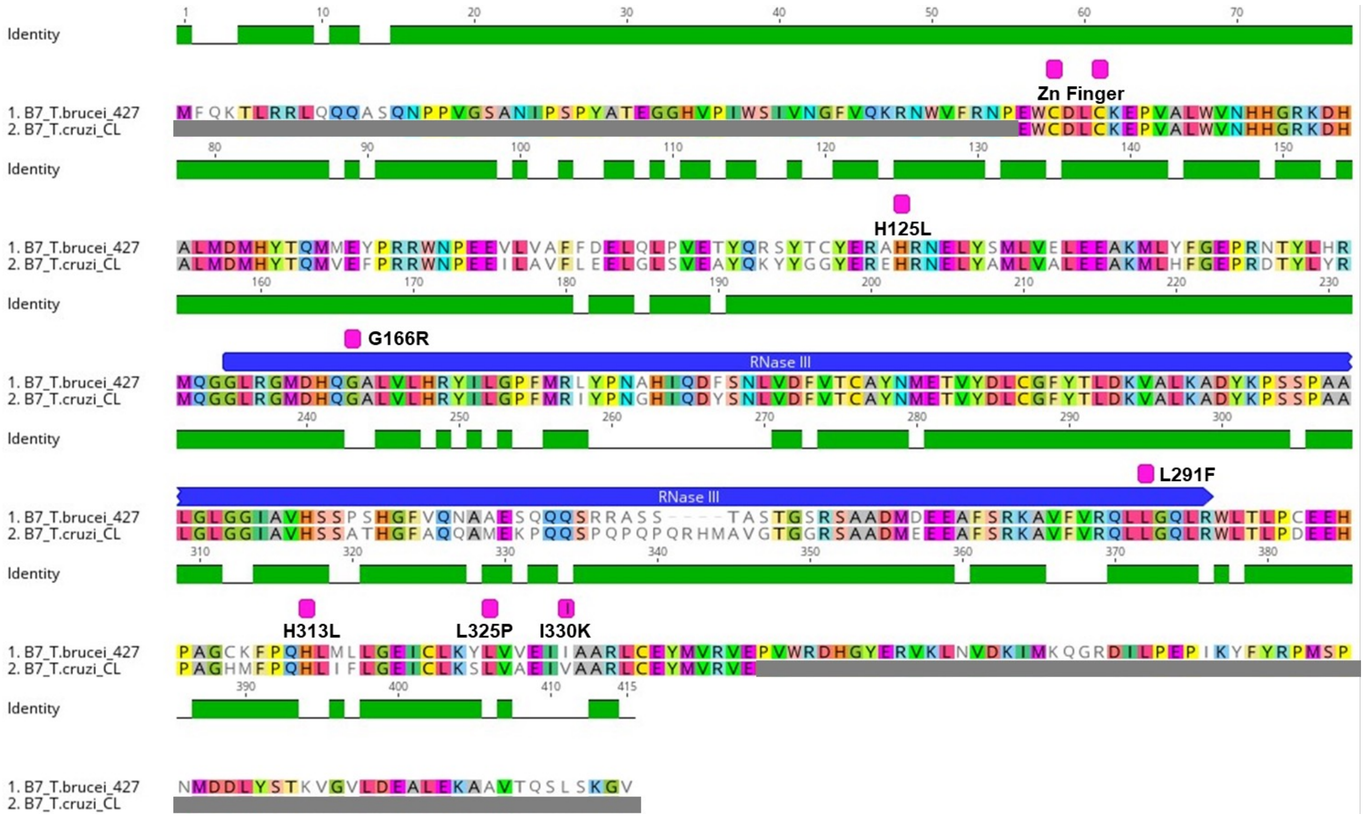

Supplementary Figure 6.

T cruzi KREPB8 (Q4CX36) Alphafold model
Deleted low and very low confidence regions at N terminus (AlphaFold pLDDT score < 70) M1-K81
Deleted regions correspond to *T. brucei* M1-A55

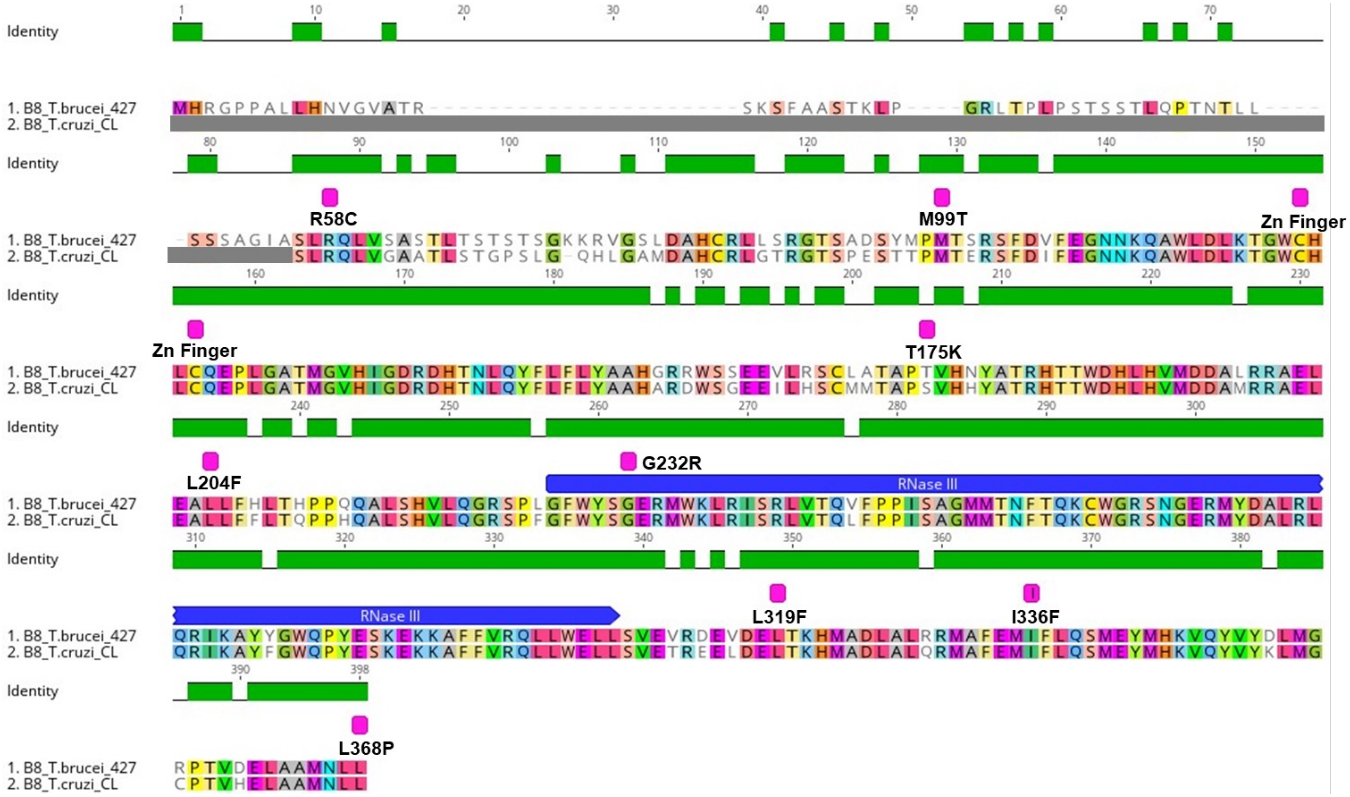

Supplementary Figure 7.

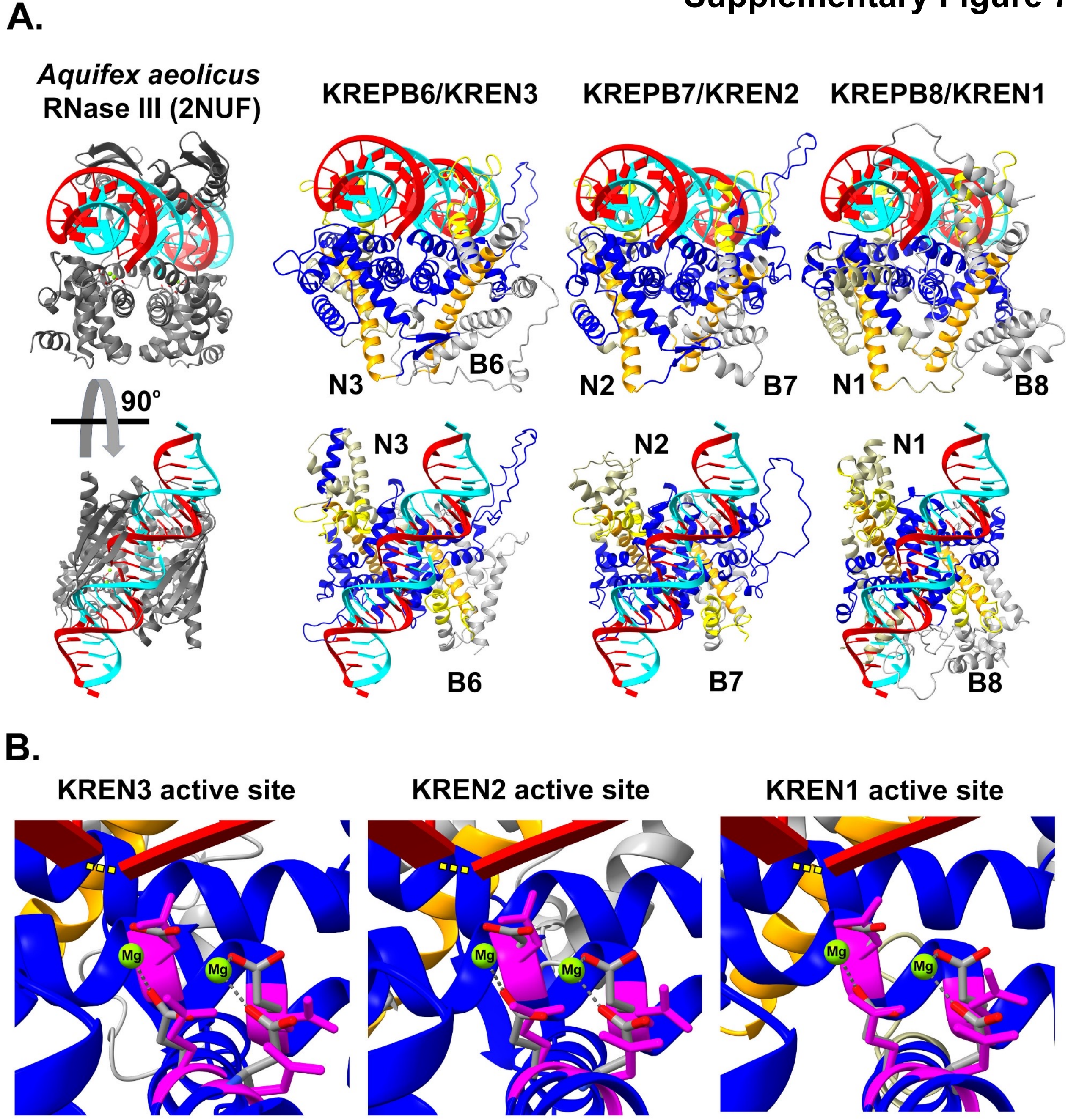

Supplementary Figure 8.

T cruzi KREN1 (Q4DA91) Alphafold model
Deleted low and very low confidence regions at N and C termini (AlphaFold pLDDT score < 70) M1-A119 and I410-M779*
Deleted regions correspond to *T. brucei* M1-V135 and I426-A818*

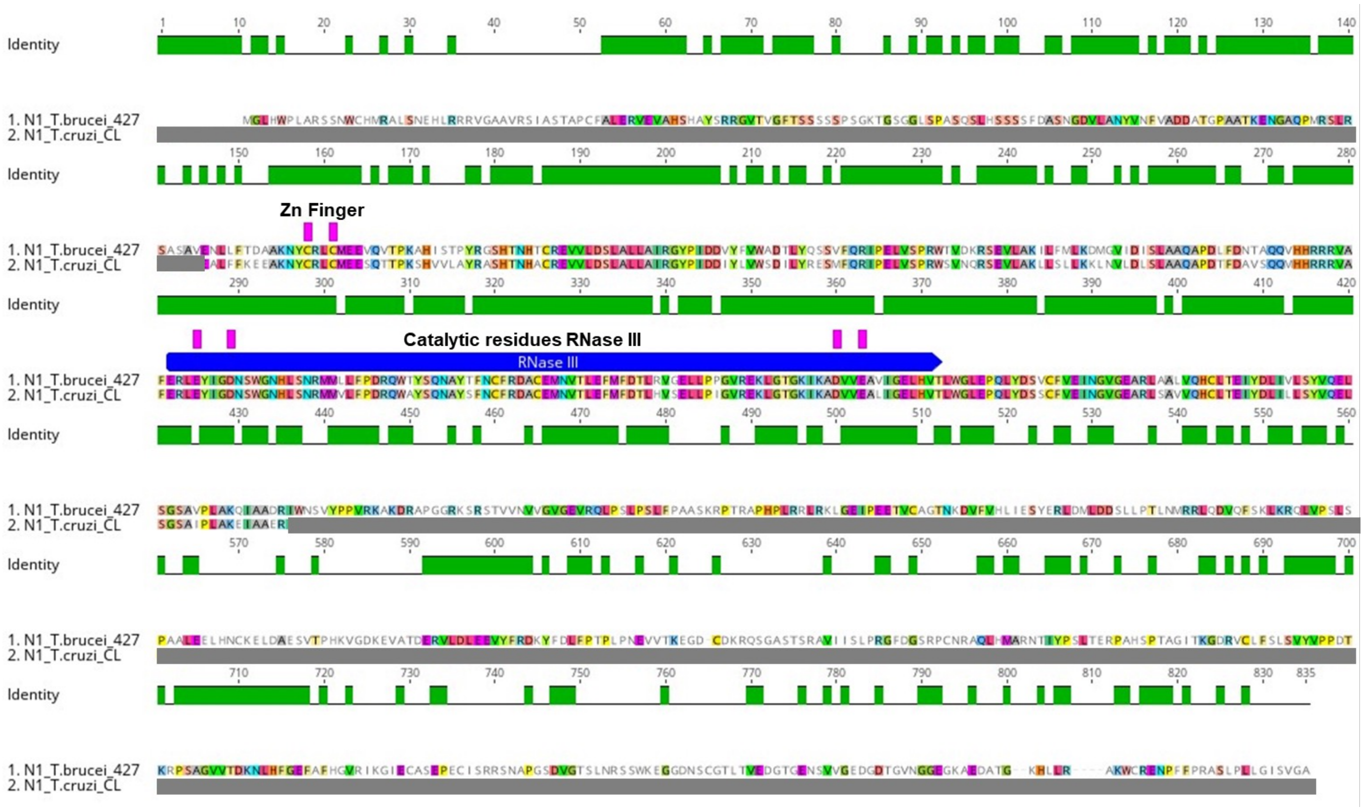

Supplementary Figure 9.

T cruzi KREN2 (Q4E082) Alphafold model
Deleted low and very low confidence regions at N and C termini (AlphaFold pLDDT score < 70) M1-F93 and A369-V532*
Deleted regions correspond to *T. brucei* M1-G95 and A375-V538*

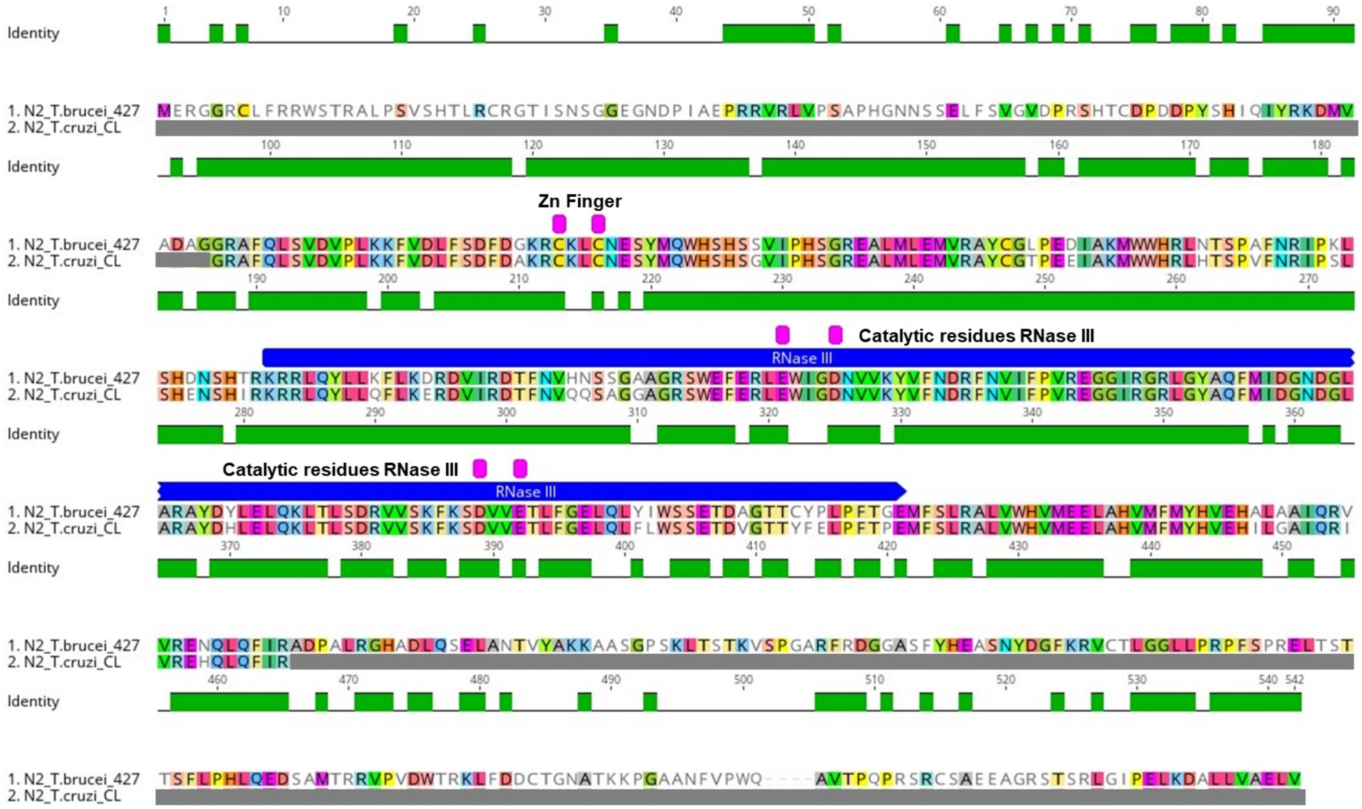

Supplementary Figure 10.

T cruzi KREN3 (Q4E095) Alphafold model
Deleted low and very low confidence regions at N and C termini (AlphaFold pLDDT score < 70) M1-M45 and S343-E593*
Deleted regions correspond to *T. brucei* M1-S45 and A343-G596*

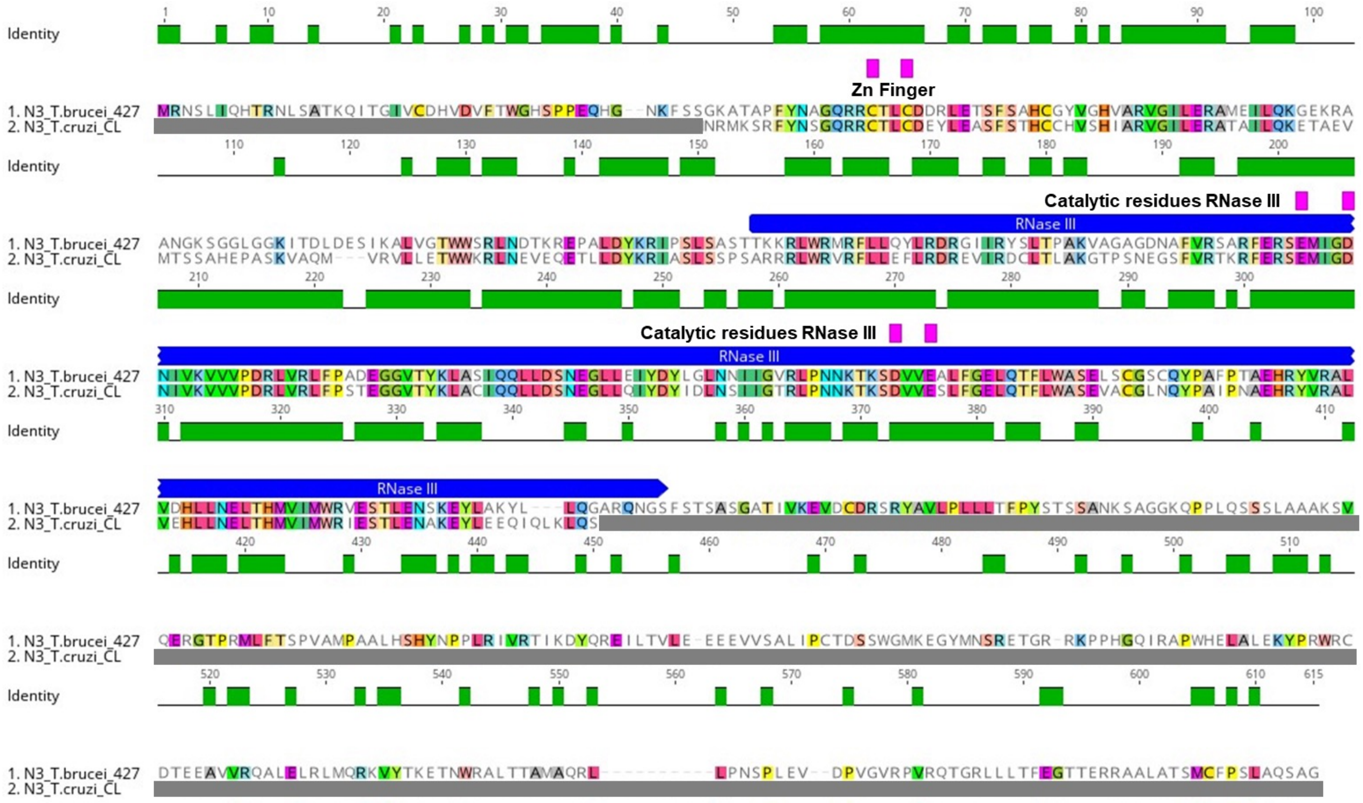

Supplementary Figure 11.

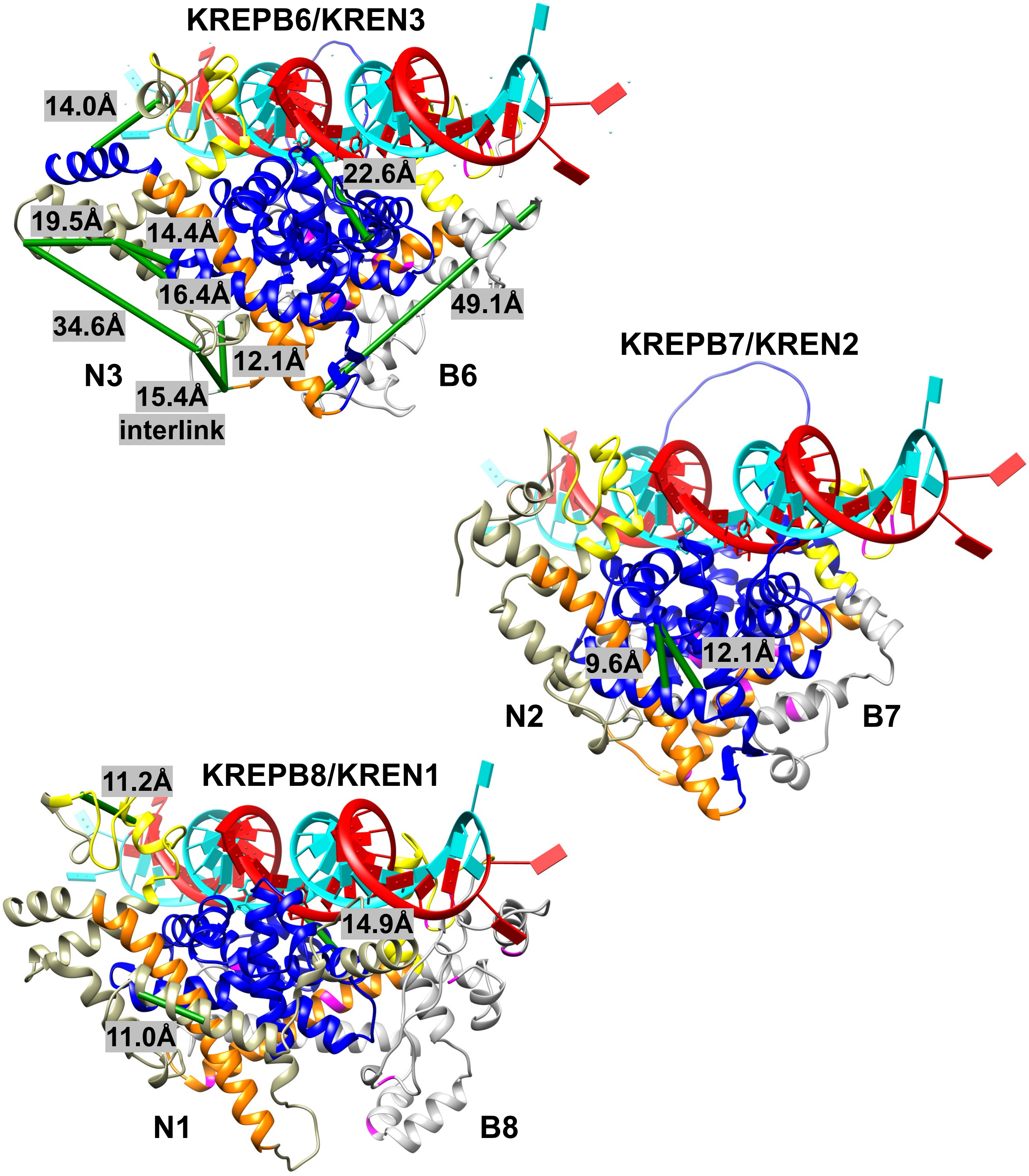

Supplementary Figure 12.

KREPB6/KREN3	KREPB7/KREN2	KREPB8/KREN1

Residues crosslinking to KREPA1:

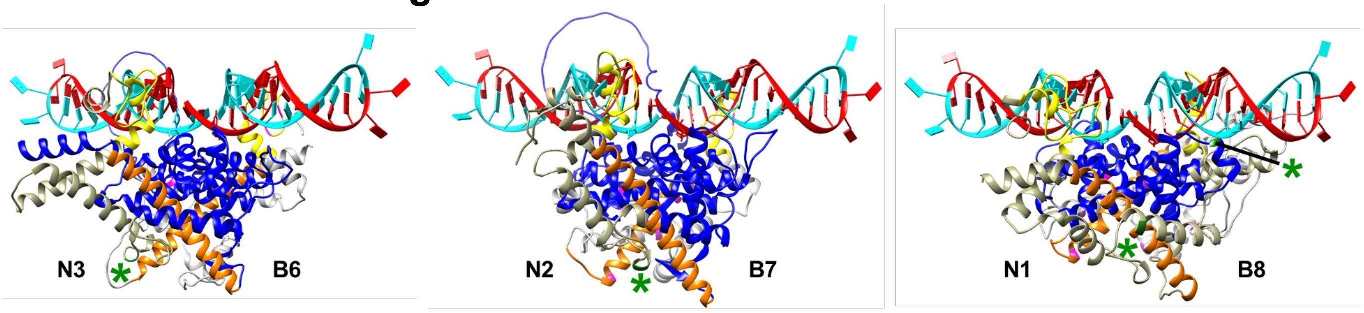

Residues crosslinking to KRET2:

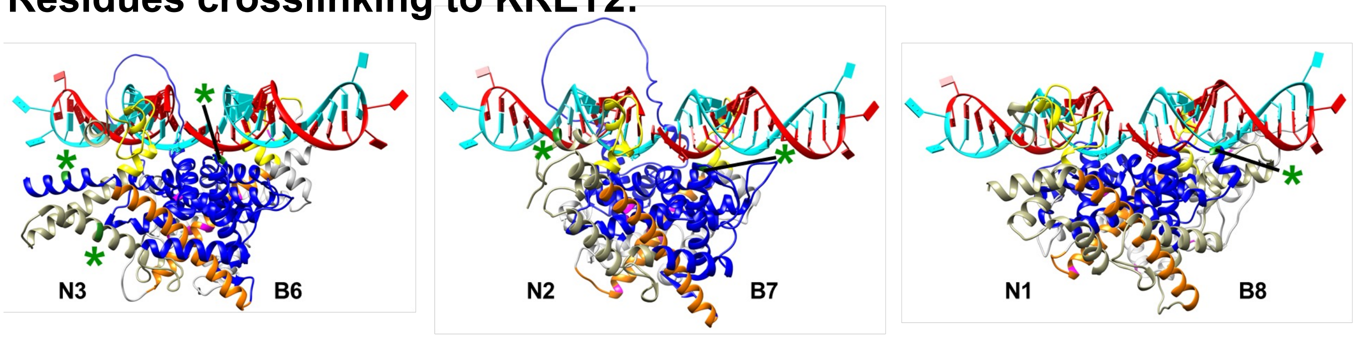

Residues crosslinking to KREPB4:

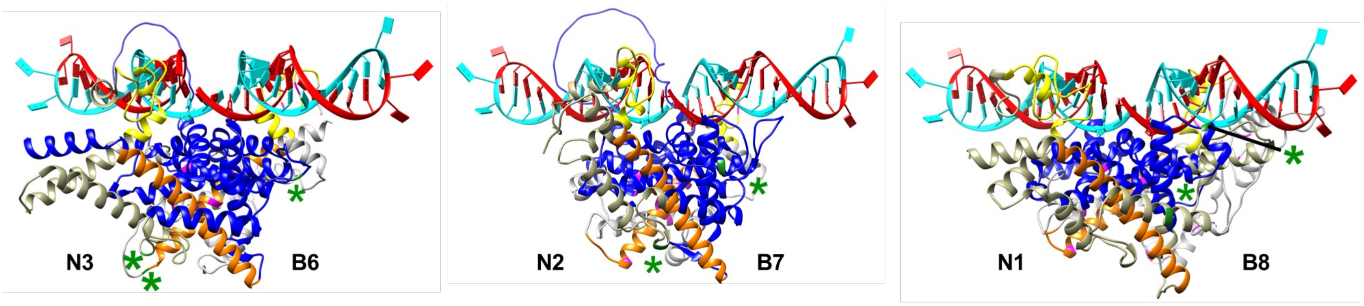

Residues crosslinking to KREX1 in KREN1:

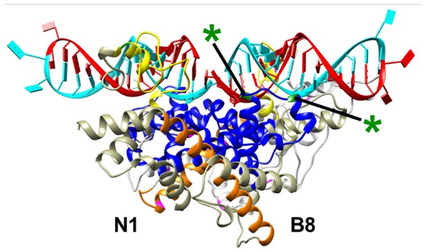

Supplementary Figure 13.

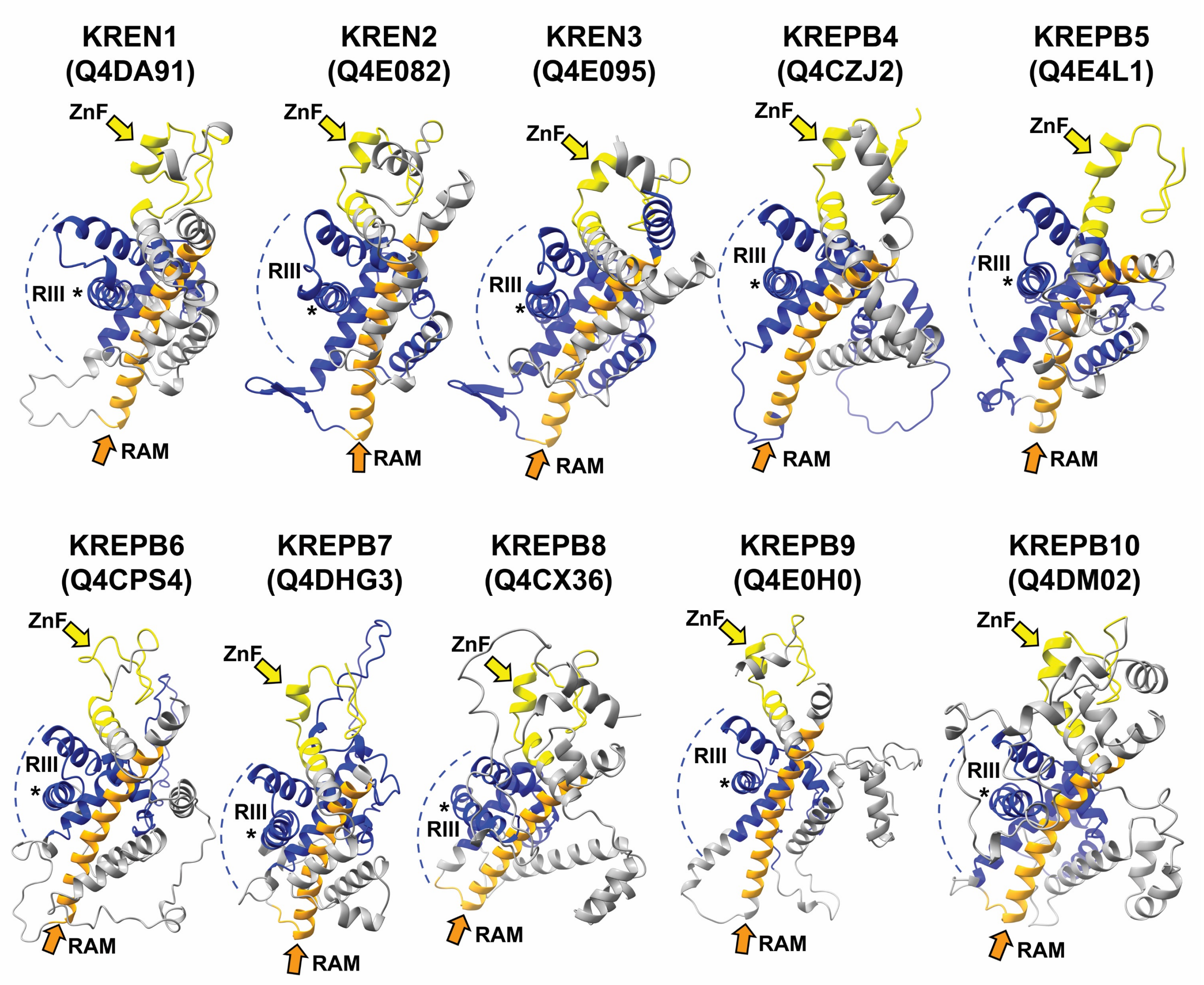

Supplementary Figure Legends

Supplementary Figure 1. Amplification of pre-, partially-, and fully-edited mRNAs in control PF 29.13 and BF SM427 cell lines by RT-PCR. Amplification was carried out using different numbers of PCR cycles as indicated. Brackets indicate gel regions that span the sizes from pre-edited (P) to fully edited (E) RT-PCR products from A6, MURF2, or RPS12, ND7-5', ND8, or CYb mRNAs. Never-edited (NE) ND4 mRNA is included as a mitochondrial mRNA control.

Supplementary Figure 2. Composition of random mutagenesis libraries for B6, B7 and B8. A considerable proportion of the sequences in each library contain a single amino acid change, and none are dominated by WT (i.e. 0 changes).

Supplementary Figure 3. MUSCLE alignment of RNase III-Associated Motif (RAM) of *T. brucei* B6, B7 and B8 and seven paralogs that also associate with RECCs. Amino acids identified as essential in B6, B7, and B8 are highlighted with red boxes and indicated by red arrows. N1, N2, N3, B4, and B5 have a potential PUF motif that we previously identified (1) which contains a conserved histidine at position 13.

Supplementary Figures 4-6. MUSCLE alignments of *T. brucei* and *T. cruzi* B6, B7, and B8. Gray bars and text define protein sequences that were removed from the Alphafold predicted *T. cruzi* protein structures due to low or very low Alphafold confidence scores. Positions of single amino acid substitutions that were characterized in this study are indicated by magenta bars, and the previously annotated RNase III motifs are highlighted by blue bars.

Supplementary Figure 7. Models of B6/N3, B7/N2, and B8/N1 dimers based on the predicted *T. cruzi* B6-B8 and N1-N3 Alphafold structures and the crystal structure of the *A. aeolicus* RNase III dimer with RNA substrate (PDB structure 2NUF). **(A)** The bottom structures are rotated 90° toward the viewer. The RECC protein ZnF motifs are yellow, RNase III motifs are blue, and RAMs are orange. Both strands of RNA are cleaved in the 2NUF structure but N1, N2 and N3 have catalytic amino acids that would only cleave the red strand and the partner B proteins are unable to cleave cyan strand. The placements of ZnF motifs in RECC structures mirror dsRBDs in 2NUF that bind substrate RNA. **(B)** Closeup detail of the active sites for N3, N2 and N1 with catalytic amino acids in magenta compared to the catalytic side chains of 2NUF in gray and red. The high degree of similarity in these catalytic residues strongly supports the quality of the Alphafold predicted structures. The Mg^{2+} ions (green) are coordinated as shown by the green dotted lines and the location the cleaved RNA strand is shown by the yellow dotted yellow line.

Supplementary Figures 8-10. MUSCLE alignments of *T. brucei* and *T. cruzi* N1, N2, and N3. Gray bars and text define protein sequences that were removed from the Alphafold predicted *T. cruzi* protein structures due to low or very low Alphafold confidence scores. Conserved cysteine residues in the ZnFs, and catalytic residues in the RNase III motifs are indicated by magenta bars. The previously annotated RNase III motifs are highlighted by blue bars.

Supplementary Figure 11. Visualization of intra- and one inter-crosslink identifications from (2) on the predicted structures and Rnt1p-based models of B6/N3, B7/N2, and B8/N1. The *T. cruzi* residues that align with crosslinked *T. brucei* residues from the previous study were identified and links between them shown in green with Cα-Cα distance measurements alongside. In most cases, the distances between crosslinked residues were <30 Å meaning that the predicted structures and models are generally consistent with the available experimental data. All other colors highlighting protein motifs and mutations are as in Figures 9 and 10.

Supplementary Figure 12. Mapping of residues that crosslink with RECC proteins A1, T2, B4, and X1 (N1 only) in (2) on the predicted structures and Rnt1p-based models of B6/N3, B7/N2, and B8/N1. The *T. cruzi* residues that align with crosslinked *T. brucei* residues from the previous study shown in green and asterisked. All other colors highlighting protein motifs and mutations are as in Figures 9 and 10.

Supplementary Figure 13. Alphafold predicted structures for *T. cruzi* RECC RNase IIIs, with indicated UniProt IDs. N- or C-terminal regions with low or very low Alphafold confidence scores not shown for clarity. The RECC protein ZnF motifs are yellow and highlighted with yellow arrows; RNase III motifs are blue with dimerization helix noted with an asterisk and highly conserved region bracketed by dashed arc, and RAMs are orange and highlighted with orange arrow. Note the broad similarities in structures among these paralogs.

Supplementary Tables

Supplementary Table 1. Full terminology and abbreviations used for RECC proteins.

Gene ID	Full name	Abbreviation	Previously annotated motifs
Tb927.3.3990	KREPB6	B6	Matrin-type C2H2 ZnF<br>RNase III (lacks catalytic residues)
Tb927.9.5630	KREPB7	B7	Matrin-type C2H2 ZnF<br>RNase III (lacks catalytic residues)

Tb927.8.5690	KREPB8	B8	Matrin-type C2H2 ZnF<br>RNase III (lacks catalytic residues)
Tb927.1.1690	KREN1	N1	Matrin-type C2H2 ZnF<br>RNase III (catalytic residues)<br>PUF
Tb927.10.5440	KREN2	N2	Matrin-type C2H2 ZnF<br>RNase III (catalytic residues)<br>PUF
Tb927.10.5320	KREN3	N3	Matrin-type C2H2 ZnF<br>RNase III (catalytic residues)<br>PUF
Tb927.11.2990	KREPB4	B4	Matrin-type C2H2 ZnF<br>RNase III (lacks catalytic residues)<br>PUF
Tb927.11.940	KREPB5	B5	Matrin-type C2H2 ZnF (divergent)<br>RNase III (lacks catalytic residues)<br>PUF
Tb927.9.4440	KREPB9	B9	Matrin-type C2H2 ZnF<br>RNase III (lacks catalytic residues)
Tb927.8.5700	KREPB10	B10	Matrin-type C2H2 ZnF<br>RNase III (lacks catalytic residues)
Tb927.2.2470	KREPA1	A1	C2H2 ZnFs<br>OB fold
Tb927.10.8210	KREPA2	A2	C2H2 ZnFs<br>OB-fold
Tb927.8.620	KREPA3	A3	C2H2 ZnFs<br>OB fold
Tb927.10.5110	KREPA4	A4	OB fold
Tb927.8.680	KREPA5	A5	OB fold
Tb927.10.5120	KREPA6	A6	OB fold
Tb927.9.4360	KREL1	L1	RNA ligase (Rnl2)
Tb927.1.3030	KREL2	L2	RNA ligase (Rnl2)
Tb927.7.1550	KRET2	T2	TUTase, PAP associated
Tb927.7.1070	KREX1	X1	5’-3’ exonuclease<br>Exo/endo/phos (EEP)
Tb927.10.3570	KREX2	X2	5’-3’ exonuclease<br>Exo/endo/phos (EEP)

Supplementary Table 2. Sequences of oligonucleotides used in this study.

Primer number	Primer Description	Primer sequence	Reference
5355	FOR oligo anneals 5' of the β-tubulin locus integration site.	GTACGCTGCTCATCTCGAAGCT	(3)
5356	REV oligo anneals 3' of ORF integrated into the β-tubulin	TTGGCCACACAACCCGGTGTTA	(3)
6391	V5-tag in pHD1344tub (PAC)GW-Cterm3V5 REV PCR primer	TCTAGATCTCGTGCTATCAAGACCGAGGA	(3)
9571	GPEET splice-acceptor site in pHD1344tub (PAC)GW-Cterm3V5 FOR sequencing primer	GCTGCACGCGCCTTCGAGT	(3)
10622	V5-tag in pHD1344tub (PAC)GW-Cterm3V5 REV sequencing primer	ACCTCGCGTAGAATCGAGAC	(3)
10150	REV oligo anneals at start of actin 3'UTR to PCR amplify ORF in pHD1344 tub GW)	GTCAACTCACAGCGACTACA	This Study
11316	FOR oligo sequencing inserts in pHD1344tub (PAC)GW-Cterm3V5	TGAAGACTTCAATTACAC	This Study
11317	REV oligo sequencing inserts in pHD1344tub (PAC)GW-Cterm3V5	GATAGGCTTACCTTCGAAC	This Study
	*att*B1 epPCR forward	ACAAGTTTGTACAAAAAAGCAG	(3)

	*att*B2 epPCR reverse	ACCACTTTGTACAAGAAAGCT	(3)
	B6 ZnF (C69A & C72A) site-directed mutagenesis	CAACTGTTAAGTGGCTCCAAGGCAAGACTGGCCCAATTCTCCGTTTCATTCCA	This Study
	B6 ZnF (C69A & C72A) site-directed mutagenesis	TGGAATGAAACGGAGAATTGGGCCAGTCTTGCCTTGGAGCCACTTAACAGTTG	This Study
	B6 C177R site-directed mutagenesis	TCAAATGTTGAATGCGAGCGTAGAGCTCCAAACGC	This Study
	B6 C177R site-directed mutagenesis	GCGTTTGGAGCTCTACGCTCGCATTCAACATTTGA	This Study
	B6 T206R site-directed mutagenesis	GGAAATAATTCCTTGAATCTCACGAGAGAACCGTGG	This Study
	B6 T206R site-directed mutagenesis	CCACGGTTCTCTCGTGAGATTCAAGGAATTATTTCC	This Study
	B6 L214S site-directed mutagenesis	GCAAATGGAAACATATTTACTGAATTTGGAAATAATTCCTTGAATGTCACG	This Study
	B6 L214S site-directed mutagenesis	CGTGACATTCAAGGAATTATTTCCAAATTCAGTAAATATGTTTCCATTTGC	This Study
	B6 T230A site-directed mutagenesis	CGAGGCTATCATTTGTGCTAAGGCGGAGATCTCCT	This Study
	B6 T230A site-directed mutagenesis	AGGAGATCTCCGCCTTAGCACAAATGATAGCCTCG	This Study
	B6 Q231H site-directed mutagenesis	TACGTCGAGGCTATCATATGTGTTAAGGCGGAGAT	This Study
	B6 Q231H site-directed mutagenesis	ATCTCCGCCTTAACACATATGATAGCCTCGACGTA	This Study
	B6 F248S site-directed mutagenesis	CTGGAAGCAACCCCTGGCTGTTACACAAGTCGAATACCG	This Study
	B6 F248S site-directed mutagenesis	CGGTATTCGACTTGTGTAACAGCCAGGGGTTGCTTCCAG	This Study
	B6 W318R site-directed mutagenesis	CACTCCAATGCCCTTCGTAATTGACCCATCACGC	This Study
	B6 W318R site-directed mutagenesis	GCGTGATGGGTCAATTACGAAGGGCATTGGAGTG	This Study
	B6 T350I site-directed mutagenesis	GAGGGCCCGACAGATGTATTCCGAAAGAACTTG	This Study
	B6 T350I site-directed mutagenesis	CAAGTTCTTTCGGAATACATCTGTCGGGCCCTC	This Study

	B6 R352W site-directed mutagenesis	AAGGAGGGCCCAACAGGTGTATTCCGAAAGAAC	This Study
	B6 R352W site-directed mutagenesis	GTTCTTTCGGAATACACCTGTTGGGCCCTCCTT	This Study
	B6 M358V site-directed mutagenesis	GACAAAAAACCACTTCCGCAAGGAGGGCCCGAC	This Study
	B6 M358V site-directed mutagenesis	GTCGGGCCCTCCTTGCGGAAGTGGTTTTTTGTC	This Study
	B6 F360S site-directed mutagenesis	CCGAAACACGACAACTAACCATTTCCGCAAGGAGGGC	This Study
	B6 F360S site-directed mutagenesis	GCCCTCCTTGCGGAAATGGTTAGTTGTCGTGTTTCGG	This Study
	B6 E365D site-directed mutagenesis	CTCAACGCGCACGATATAATCCGAAACACGACAAAAA	This Study
	B6 E365D site-directed mutagenesis	TTTTTGTCGTGTTTCGGATTATATCGTGCGCGTTGAG	This Study
	B7 ZnF (C58A & C61A) site-directed mutagenesis	CAATGCCACAGGTTCTTTGGCAAGGTCAGCCCACTCAGGGTTACGGAAC	This Study
	B7 ZnF (C58A & C61A) site-directed mutagenesis	GTTCCGTAACCCTGAGTGGGCTGACCTTGCCAAAGAACCTGTGGCATTG	This Study
	B7 L60H site-directed mutagenesis	CCTGAGTGGTGTGACCATTGCAAAGAACCTGTG	This Study
	B7 L60H site-directed mutagenesis	CACAGGTTCTTTGCAATGGTCACACCACTCAGG	This Study
	B7 W68S site-directed mutagenesis	GCAAAGAACCTGTGGCATTGTCGGTGAACCATC	This Study
	B7 W68S site-directed mutagenesis	GATGGTTCACCGACAATGCCACAGGTTCTTTGC	This Study
	B7 K75T site-directed mutagenesis	GAACCATCACGGTCGTACGGACCACGCCT	This Study
	B7 K75T site-directed mutagenesis	AGGCGTGGTCCGTACGACCGTGATGGTTC	This Study
	B7 H83L site-directed mutagenesis	CACGCCTTAATGGACATGCTCTATACACAAATGATGGAA	This Study
	B7 H83L site-directed mutagenesis	TTCCATCATTTGTGTATAGAGCATGTCCATTAAGGCGTG	This Study

	B7 H125L site-directed mutagenesis	CATGTTACGAACGTGCCCTTAGGAATGAGCTTTACTC	This Study
	B7 H125L site-directed mutagenesis	GAGTAAAGCTCATTCCTAAGGGCACGTTCGTAACATG	This Study
	B7 L290S site-directed mutagenesis	GCTGTTTTTGTTAGACAGTCGTTAGGTCAACTGCGATGG	This Study
	B7 L290S site-directed mutagenesis	CCATCGCAGTTGACCTAACGACTGTCTAACAAAAACAGC	This Study
	B7 L291F site-directed mutagenesis	GTTTTTGTTAGACAGTTGTTTGGTCAACTGCGATGGTTG	This Study
	B7 L291F site-directed mutagenesis	CAACCATCGCAGTTGACCAAACAACTGTCTAACAAAAAC	This Study
	B7 H313L site-directed mutagenesis	TTGTAAATTCCCCCAACTCCTGATGCTCTTGGGTG	This Study
	B7 H313L site-directed mutagenesis	CACCCAAGAGCATCAGGAGTTGGGGGAATTTACAA	This Study
	B7 L325P site-directed mutagenesis	GAGATTTGCCTCAAGTATCCTGTGGTGGAGATAATAGCG	This Study
	B7 L325P site-directed mutagenesis	CGCTATTATCTCCACCACAGGATACTTGAGGCAAATCTC	This Study
	B7 I330K site-directed mutagenesis	AGTATCTTGTGGTGGAGATAAAAGCGGCACGCC	This Study
	B7 I330K site-directed mutagenesis	GGCGTGCCGCTTTTATCTCCACCACAAGATACT	This Study
	B8 ZnF (C123A & C126A) site-directed mutagenesis	ATCTCAAGACTGGCTGGGCCCATCTCGCCCAAGAACCACTAGGTG	This Study
	B8 ZnF (C123A & C126A) site-directed mutagenesis	CACCTAGTGGTTCTTGGGCGAGATGGGCCCAGCCAGTCTTGAGAT	This Study
	B8 R58C site-directed mutagenesis	CGCTGACGAGCTGGCACAGAGAAGCTATTCC	This Study
	B8 R58C site-directed mutagenesis	GGAATAGCTTCTCTGTGCCAGCTCGTCAGCG	This Study
	B8 M99T site-directed mutagenesis	TCAGCCGATAGTTACATGCCAACGACGTCACGCTC	This Study

	B8 M99T site-directed mutagenesis	GAGCGTGACGTCGTTGGCATGTAACTATCGGCTGA	This Study
	B8 V106D site-directed mutagenesis	CTTGTTGTTACCTTCAAAATCATCGAAAGAGCGTGACGT	This Study
	B8 V106D site-directed mutagenesis	ACGTCACGCTCTTTCGATGATTTTGAAGGTAACAACAAG	This Study
	B8 T175K site-directed mutagenesis	GTTGTGCACCTTCGGAGCGGTGGCG	This Study
	B8 T175K site-directed mutagenesis	CGCCACCGCTCCGAAGGTGCACAAC	This Study
	B8 L204F site-directed mutagenesis	GTCAGATGGAAAAGAAACGCCTCCAACTCAGCG	This Study
	B8 L204F site-directed mutagenesis	CGCTGAGTTGGAGGCGTTTCTTTTCCATCTGAC	This Study
	B8 H207Y site-directed mutagenesis	GTGGGTGCGTCAGATAGAAAAGAAGCGCCTC	This Study
	B8 H207Y site-directed mutagenesis	GAGGCGCTTCTTTTCTATCTGACGCACCCAC	This Study
	B8 L319F site-directed mutagenesis	CGCCATGTGTTTTGTAAATTCATCTACCTCGTCACG	This Study
	B8 L319F site-directed mutagenesis	CGTGACGAGGTAGATGAATTTACAAAACACATGGCG	This Study
	B8 I336F site-directed mutagenesis	CCATTGACTGCAAGAAGAACATCTCAAACGCCATACG	This Study
	B8 I336F site-directed mutagenesis	CGTATGGCGTTTGAGATGTTCTTCTTGCAGTCAATGG	This Study
	B8 L368P site-directed mutagenesis	AAGAAAGCTGGGTCCGGCAAATTCATTGCTGCAAG	This Study
	B8 L368P site-directed mutagenesis	CTTGCAGCAATGAATTTGCCGGACCCAGCTTTCTT	This Study
6204	FOR MURF2 pre-edited/edited RT-PCR	ATAGAAAGGTATATAATCTATAATG	(4)
4934	REV MURF2 pre-edited/edited RT-PCR	AATATAAAATCTAGATCAAACCATCACA	(4)
3704	FOR A6 pre-edited/edited RT-PCR	AAAAATAAGTATTTTGATATTATTAAAG	(4,5)

3580	REV A6 pre-edited/edited RT-PCR	TATTATTAACTTATTTGATC	(4,5)
3619	FOR RPS12 pre-edited/edited RT-PCR	CTAATACACTTTTGATAACAAAC	(5,6)
3620	REV RPS12 pre-edited/edited RT-PCR	AAAAACATATCTTATATCTAAA	(5,6)
3706	FOR ND4 pre-edited/edited RT-PCR	TGTGTGACTACCAGAGAT	(4,5)
3707	REV ND4 pre-edited/edited RT-PCR	ATCCTATACCCGTGTGTA	(4,5)
3705	FOR ND7-5’ pre-edited/edited RT-PCR	ATGACTACATGATAAGTA	(5)
3601	REV ND7-5’ pre-edited/edited RT-PCR	CGGAAGACATTGTTCTACAC	(5)
3623	FOR ND8 pre-edited/edited RT-PCR	CAATTTAATAATTTTAAGTTTTGG	This Study
3624	REV ND8 pre-edited/edited RT-PCR	TAGTCAAAATTTAATTTCACCGTG	This Study
4311	FOR CYb pre-edited/edited RT-PCR	GTTAAGAATAATGGTTATAAATTTTATAT	This Study
4931	REV CYb pre-edited/edited RT-PCR	CCCATATATTCTATATAAACAACCTGACA	(7)

Supplementary Table 3. Antibodies used in this study.

Antibody name	Raised in	Dilution	Source
V5 Epitope Tag Monoclonal Antibody	Mouse	1/5000 WB	ThermoFisher Scientific R960-25
HA Epitope Tag (2-2.1.14) Monoclonal Antibody	Mouse	1/200 WB	ThermoFisher Scientific 26183
KREPA1 Monoclonal Antibody	Mouse	1/25 WB	(8) P4D8-F6
KREPA2 Monoclonal Antibody	Mouse	1/12.5 WB	(8) P1H3-D7

KREL1 Monoclonal Antibody	Mouse	1/50 WB	(8) P3C1-G2
KREPA3 Monoclonal Antibody	Mouse	1/25 WB	(8) P3C12-B6
mtHSP70 Monoclonal Antibody	Mouse	1/1000 WB	(9) mAb78

Supplementary Table 4. Gene IDs for sequences used in multi-species alignments of RECC endonuclease paralogs. n/a = not applicable.

Species	KREN1	KREN2	KREN3	KREPB4
Angomonas deanei	ADEAN_000267500	ADEAN_000164000	ADEAN_000162400	ADEAN_000309400
Blechomonas ayalai	Baya_032_0140	Baya_056_0190	Baya_056_0080	Baya_003_0800
Bodo saltans	BSAL_24845	n/a	n/a	BSAL_54985
Crithidia fasciculata	CFAC1_180016200	CFAC1_250018800	CFAC1_250016900	CFAC1_230023500
Leishmania aethiopica	LAEL147_000302700	LAEL147_000818500	LAEL147_000817100	LAEL147_000687400
Leishmania arabica	LARLEM1108_200014800	LARLEM1108_360015700	LARLEM1108_360014700	LARLEM1108_330024800
Leishmania braziliensis	LbrM.20.5080	LbrM.35.1180	LbrM.35.1040	LbrM.33.2.002250
Leishmania donovani	LdBPK_200910.1	LdBPK_361110.1	LdBPK_360980.1	LdBPK_332090.1
Leishmania enriettii	LENLEM3045_200014500	LENLEM3045_360016300	LENLEM3045_360015100	LENLEM3045_330027900
Leishmania major	LmjF.20.0900	LmjF.36.1050	LmjF.36.0920	LmjF.33.1970
Leishmania mexicana	LmxM.20.0900	LmxM.36.1050	LmxM.36.0920	LmxM.32.1970
Leptomonas pyrrhocoris	LpyrH10_25_1020	LpyrH10_09_1250	LpyrH10_09_1080	LpyrH10_07_2800
Leptomonas seymouri	Lsey_0119_0100	Lsey_0117_0190	Lsey_0028_0020	Lsey_0022_0130
Leishmania tarentolae	LtaP20.0880	LtaP36.1050	LtaP36.0940	LtaP33.2160
Trypanosoma brucei	Tb427.01.1690	Tb427.10.5440	Tb427.10.5320	Tb427tmp.02.0490
Trypanosoma congolense	TcIL3000_0_54390	TcIL3000_10_4590	TcIL3000_10_4470	TcIL3000.11.2750
Trypanosoma cruzi	TcCLB.509991.90	TcCLB.506679.210	TcCLB.506679.90	TcCLB.507773.30
Trypanosoma rangeli	TRSC58_03894	TRSC58_05826	TRSC58_00961	TRSC58_00967

Species	KREPB5	KREPB6	KREPB7	KREPB8
Angomonas deanei	*ADEAN_000283300*	n/a	*ADEAN_000949400*	*ADEAN_000008800*
Blechomonas ayalai	*Baya_102_0210*	*Baya_040_0350*	*Baya_049_0160*	*Baya_147_0120*
Bodo saltans	*BSAL_46395*	n/a	*BSAL_28040*	*BSAL_15080*
Crithidia fasciculata	*CFAC1_230049400*	*CFAC1_200021100*	*CFAC1_240045200*	*CFAC1_120009900*
Leishmania aethiopica	*LAEL147_000457600*	*LAEL147_000530600*	*LAEL147_000220700*	*LAEL147_000227800*
Leishmania arabica	*LARLEM1108_270008200*	*LARLEM1108_290021500*	*LARLEM1108_150018500*	*LARLEM1108_160008300*
Leishmania braziliensis	*LbrM.27.2.000350*	*LbrM.29.1520*	*LbrM.15.1210*	*LbrM.16.0340*
Leishmania donovani	*LdBPK_270350.1*	*LdBPK_291540.1*	*LdBPK_151290.1*	*LdBPK_160340.1*
Leishmania enriettii	*LENLEM3045_270008800*	*LENLEM3045_290020900*	*LENLEM3045_150018600*	*LENLEM3045_160008900*
Leishmania major	*LmjF.27.0340*	*LmjF.29.1430*	*LmjF.15.1270*	*LmjF.16.0330*
Leishmania mexicana	*LmxM.27.0340*	*LmxM.08_29.1430*	*LmxM.15.1270*	*LmxM.16.0330*
Leptomonas pyrrhocoris	*LpyrH10_35_0370*	*LpyrH10_08_1720*	*LpyrH10_26_0440*	*LpyrH10_15_0390*
Leptomonas seymouri	*Lsey_0115_0090*	*Lsey_0002_0300*	*Lsey_0048_0040*	*Lsey_0026_0120*
Leishmania tarentolae	*LtaP27.0330*	*LtaP29.1580*	*LtaP15.1180*	*LtaP16.0330*
Trypanosoma brucei	*Tb427_110014100*	*Tb427.03.3990*	*Tb427tmp.160.4130*	*Tb427.08.5690*
Trypanosoma congolense	*TcIL3000.A.H_000870300*	*TcIL3000_3_2640*	*TcIL3000_9_1880*	*TcIL3000_8_5460*
Trypanosoma cruzi	*C4B63_19g190*	*TcCLB.509623.10*	*TcCLB.506445.80*	*TcCLB.509253.40*
Trypanosoma rangeli	n/a	*TRSC58_02464*	*TRSC58_00794*	*TRSC58_01960*

Supplementary Table 5. Compositions of B6, B7, and B8 mutant allele libraries following selection in *E. coli* to remove nonsense and frameshift mutations.

Library	Average aa changes per ORF	Mode aa changes per ORF	Range aa changes per ORF	% ORFs with single change	% ORFs encoding WT protein	Number (%) aa in protein with changes
B6	2.06	1	0-7	31	12.5	114/438 (26)
B7	2.23	1	0-9	29	26	103/411 (25)
B8	1.96	1	0-6	23	20	53/368 (14)

Supplementary Table 6. Mutant allele library transfections and complementation screening in *T. brucei*. Loss of tet-induced WT expression in the CN cell lines results in the exclusive expression of mutant alleles, leading to growth defects if mutant alleles cannot complement function. Growth defects are assessed via an Alamar Blue colorimetric assay, in which cultures without growth defects are pink, cultures with strong defects are blue, and cultures with intermediate growth defects are purple.

			Number (%) of total cell lines with:	
Library	Number *T. brucei* cell lines obtained	Total number (%) of cell lines with grow defect	Strong growth defect	Intermediate growth defect
B6	833	215 (26%)	182 (22%)	33 (4%)
B7	835	91 (11%)	46 (6%)	45 (5%)
B8	933	275 (29%)	115 (12%)	160 (17%)

Supplementary Table 7. MUSCLE alignment of non-complementing B6, B7, and B8 single amino acid substitutions with corresponding residues in 18 kinetoplastid species. The order of the species is: 1. *T. brucei* 427, 2. *T. congolense*, 3. *T. cruzi* strain CL Brener, 4. *T. rangeli*, 5. *Leishmania aethiopica*, 6. *L. arabica*, 7. *L. braziliensis*, 8. *L. donovani*, 9. *L. enriettii*, 10. *L. major*, 11. *L. mexicana*, 12. *Leptomonas pyrrhocoris*, 13. *Leptomonas seymouri*, 14. *L. tarentolae*, 15. *Angomonas deanei*, 16. *Blechomonas ayalai*, 17. *Bodo saltans*, 18. *Crithidia fasciculata*.

* = Jensen-Shannon divergence score for the position corresponding to the indicated *T. brucei* residue calculated as in (10).

B6:

Alignment column number	Divergence Score*	Alignment column (Species 1 to 16-no *A. deanei* or *B. saltans* orthologs)	T. brucei 427 residue	Substitution
270	0.69708	CCCCLLLLLLLLLLCL	C177	C>R
308	0.68882	TTTTLLLLLLLLLLML	T206	T>R
316	0.68539	LLLLLLLLLLLMLLLL	L214	L>S
332	0.68530	TTTTSSLSLSSTTSSL	T230	T>A
333	0.70043	QQQQQQQQQQQSAQQA	Q231	Q>H
350	0.59479	LLLLTTMTITTLLALF	F248	F>S

443	0.83774	WWWWWWWWWWWWWWWW	W318	W>R
479	0.71775	TTTTAAAATAAAAATA	T350	T>I
481	0.78622	RRRRRRRRRRRRRRRR	R352	R>W
487	0.70281	MMMMLLLLLLLLLLML	M358	M>V
489	0.74150	FFFFFFFFFFFYYFFF	F360	F>S
494	0.79363	EEEEEEEEEEEEEEEE	E365	E>D
Mean score	0.67602			
SD score	0.06084			

B7:

Alignment column number	Divergence Score*	Alignment column (Species 1 to 18)	T. brucei 427 residue	Substitution
90	0.80710	LLLLLLLLLLLLLLLLLL	L60	L>H
98	0.82584	WWWWWWWWWWWWWWWWWW	W68	W>S
105	0.82389	KKKKKKKKKKKKKKKKKK	K75	K>T
113	0.83785	HHHHHHHHHHHHHHHHHH	H83	H>L
156	0.66444	HHHHGGGGGGGNNGHRRG	H125	H>L
349	0.71660	LLLLLLLLLILVVLLILI	L290	L>S
350	0.78457	LLLLLLLLLLLLLLLLLL	L291	L>F
372	0.78647	HHHHHHHHHHHHHHHYHH	H313	H>L
384	0.74250	LLLLLLLLLLLLLLLLLL	L325	L>P
389	0.67960	IIVIIIIIIIIVVIAIIV	I330	I>K
Mean score	0.76689			
SD score	0.06264			

B8:

Alignment column number	Divergence Score*	Alignment column (Species 1 to 18)	T. brucei 427 residue	Substitution
84	0.57969	RRRRYYYSSYYRRHQQRA	R58	R>C
132	0.57723	MLMMLLLLLLLFFLYYFF	M99	M>T
139	0.69692	VVIIVVMVMVVIIVIIMI	V106	V>D
309	0.62396	TASSSSSSSSSSSSSPHA	T175	T>K
338	0.74231	LLLLLLLLLLLLLLLLCL	L204	L>F
341	0.71646	HHFFYYYYYYYYYYHHRY	H207	H>Y
453	0.62047	LLLFEEEEEEETTELYTT	L319	L>F
470	0.76053	IVIVVVVVVVVVIVVVII	I336	I>F
502	0.74014	LLLLLLLLLLLLLLLLML	L368	L>P
Mean score	0.67308			
SD score	0.07287			

Supplementary Table 8. KREPB6 amino acid sequence identity heatmap (see Supplementary_Tables_8_9_10.xlsx).

Supplementary Table 9. KREPB7 amino acid sequence identity heatmap (see Supplementary_Tables_8_9_10.xlsx).

Supplementary Table 10. KREPB8 amino acid sequence identity heatmap (see Supplementary_Tables_8_9_10.xlsx).

Supplementary References

1. Carnes, J., Schnaufer, A., McDermott, S.M., Domingo, G., Proff, R., Steinberg, A.G., Kurtz, I. and Stuart, K. (2012) Mutational analysis of *Trypanosoma brucei* editosome proteins KREPB4 and KREPB5 reveals domains critical for function. *Rna*, **18**, 1897-1909.

2. McDermott, S.M., Luo, J., Carnes, J., Ranish, J.A. and Stuart, K. (2016) The Architecture of *Trypanosoma brucei* editosomes. *Proceedings of the National Academy of Sciences of the United States of America*, **113**, E6476-E6485.
3. McDermott, S.M., Carnes, J. and Stuart, K. (2015) Identification by Random Mutagenesis of Functional Domains in KREPB5 That Differentially Affect RNA Editing between Life Cycle Stages of *Trypanosoma brucei*. *Molecular and cellular biology*, **35**, 3945-3961.
4. Guo, X., Ernst, N.L., Carnes, J. and Stuart, K.D. (2010) The zinc-fingers of KREPA3 are essential for the complete editing of mitochondrial mRNAs in *Trypanosoma brucei*. *PloS one*, **5**, e8913.
5. Schnaufer, A., Panigrahi, A.K., Panicucci, B., Igo, R.P., Jr., Wirtz, E., Salavati, R. and Stuart, K. (2001) An RNA ligase essential for RNA editing and survival of the bloodstream form of *Trypanosoma brucei*. *Science*, **291**, 2159-2162.
6. Madina, B.R., Kumar, V., Metz, R., Mooers, B.H., Bundschuh, R. and Cruz-Reyes, J. (2014) Native mitochondrial RNA-binding complexes in kinetoplastid RNA editing differ in guide RNA composition. *Rna*, **20**, 1142-1152.
7. Carnes, J., Trotter, J.R., Ernst, N.L., Steinberg, A. and Stuart, K. (2005) An essential RNase III insertion editing endonuclease in *Trypanosoma brucei*. *Proceedings of the National Academy of Sciences of the United States of America*, **102**, 16614-16619.
8. Panigrahi, A.K., Gygi, S.P., Ernst, N.L., Igo, R.P., Jr., Palazzo, S.S., Schnaufer, A., Weston, D.S., Carmean, N., Salavati, R., Aebersold, R. *et al.* (2001) Association of two novel proteins, TbMP52 and TbMP48, with the *Trypanosoma brucei* RNA editing complex. *Molecular and cellular biology*, **21**, 380-389.
9. Allen, T.E., Heidmann, S., Reed, R., Myler, P.J., Goringer, H.U. and Stuart, K.D. (1998) Association of guide RNA binding protein gBP21 with active RNA editing complexes in Trypanosoma brucei. *Molecular and cellular biology*, **18**, 6014-6022.
10. Capra, J.A. and Singh, M. (2007) Predicting functionally important residues from sequence conservation. *Bioinformatics*, **23**, 1875-1882.

Matchmaker modelling parameters

5T16 models

KREN1:

Matchmaker 5T16, chain A (#0) with Tcruzi_KREN1_AF_mod_loops.pdb, chain A (#1), sequence alignment score = 123.8
with these parameters:

chain pairing: ss
Needleman-Wunsch using BLOSUM-62
ss fraction: 0.3
gap open (HH/SS/other) 18/18/6, extend 1
ss matrix: (O, S): -6 (H, O): -6 (H, H): 6 (S, S): 6 (H, S): -9 (O, O): 4
iteration cutoff: 2

RMSD between 37 pruned atom pairs is 0.951 angstroms; (across all 163 pairs: 20.055)

KREN2:

KREN3:

Matchmaker 5T16, chain A (#0) with Tcruzi_KREN3_AF_mod_loops.pdb, chain A (#2), sequence alignment score = 152.6
with these parameters:

chain pairing: ss
Needleman-Wunsch using BLOSUM-62
ss fraction: 0.3
gap open (HH/SS/other) 18/18/6, extend 1
ss matrix: (O, S): -6 (H, O): -6 (H, H): 6 (S, S): 6 (H, S): -9 (O, O): 4
iteration cutoff: 2

RMSD between 48 pruned atom pairs is 0.978 angstroms; (across all 179 pairs: 16.852)

2NUF models

KREN1:

Matchmaker TcRECC_RIII_2NUF_modeled.pdb, chain A (#1.1; 2NUF) with TcRECC_RIII_2NUF_modeled.pdb, chain A (#1.2; TcKREN1), sequence alignment score = 126.4
with these parameters:

chain pairing: ss
Needleman-Wunsch using BLOSUM-62
ss fraction: 0.3
gap open (HH/SS/other) 18/18/6, extend 1
ss matrix: (O, S): -6 (H, O): -6 (H, H): 6 (S, S): 6 (H, S): -9 (O, O): 4
iteration cutoff: 2

RMSD between 62 pruned atom pairs is 1.152 angstroms; (across all 148 pairs: 10.064)

KREN2:

Matchmaker TcRECC_RIII_2NUF_modeled.pdb, chain A (#1.1; 2NUF) with TcRECC_RIII_2NUF_modeled.pdb, chain A (#1.3; TcKREN2), sequence alignment score = 162.1
with these parameters:

chain pairing: ss
Needleman-Wunsch using BLOSUM-62

ss fraction: 0.3
gap open (HH/SS/other) 18/18/6, extend 1
ss matrix: (O, S): -6 (H, O): -6 (H, H): 6 (S, S): 6 (H, S): -9 (O, O): 4
iteration cutoff: 2

RMSD between 58 pruned atom pairs is 1.052 angstroms; (across all 157 pairs: 13.149)

KREN3:
Matchmaker TcRECC_RIII_2NUF_modeled.pdb, chain A (#1.1; 2NUF) with TcRECC_RIII_2NUF_modeled.pdb, chain A (#1.4; TcKREN3), sequence alignment score = 174.6 with these parameters:
chain pairing: ss
Needleman-Wunsch using BLOSUM-62
ss fraction: 0.3
gap open (HH/SS/other) 18/18/6, extend 1
ss matrix: (O, S): -6 (H, O): -6 (H, H): 6 (S, S): 6 (H, S): -9 (O, O): 4
iteration cutoff: 2
RMSD between 28 pruned atom pairs is 1.246 angstroms; (across all 163 pairs: 11.051)

KREPB6:
Matchmaker TcRECC_RIII_2NUF_modeled.pdb, chain B (#1.1; 2NUF) with TcRECC_RIII_2NUF_modeled.pdb, chain A (#1.5; TcKREPB6), sequence alignment score = 106.6 with these parameters:
chain pairing: ss
Needleman-Wunsch using BLOSUM-62
ss fraction: 0.3
gap open (HH/SS/other) 18/18/6, extend 1
ss matrix: (O, S): -6 (H, O): -6 (H, H): 6 (S, S): 6 (H, S): -9 (O, O): 4
iteration cutoff: 2
RMSD between 38 pruned atom pairs is 1.256 angstroms; (across all 144 pairs: 12.575)

KREPB7:
Matchmaker TcRECC_RIII_2NUF_modeled.pdb, chain B (#1.1; 2NUF) with TcRECC_RIII_2NUF_modeled.pdb, chain A (#1.6; TcKREPB7), sequence alignment score = 87.2 with these parameters:
chain pairing: ss
Needleman-Wunsch using BLOSUM-62
ss fraction: 0.3
gap open (HH/SS/other) 18/18/6, extend 1
ss matrix: (O, S): -6 (H, O): -6 (H, H): 6 (S, S): 6 (H, S): -9 (O, O): 4
iteration cutoff: 2
RMSD between 35 pruned atom pairs is 1.122 angstroms; (across all 144 pairs: 12.761)

KREPB8:
Matchmaker TcRECC_RIII_2NUF_modeled.pdb, chain B (#1.1; 2NUF) with TcRECC_RIII_2NUF_modeled.pdb, chain A (#1.7; TcKREPB8), sequence alignment score = 153.2 with these parameters:
chain pairing: ss
Needleman-Wunsch using BLOSUM-62

ss fraction: 0.3
gap open (HH/SS/other) 18/18/6, extend 1
ss matrix: (O, S): -6 (H, O): -6 (H, H): 6 (S, S): 6 (H, S): -9 (O, O): 4
iteration cutoff: 2

RMSD between 45 pruned atom pairs is 1.214 angstroms; (across all 150 pairs: 9.502)
